# Tunable Broadband Emission via Self‐Trapped Excitons and Mn^2+^ Energy Transfer in a 0D Hybrid Manganese Bromide

**DOI:** 10.1002/smll.202504786

**Published:** 2025-07-26

**Authors:** Paulina Peksa, Maciej Ptak, Mateusz Dyksik, Alessandro Surrente, Michał Baranowski, Dawid Drozdowski, Anna Gągor, Julia Osmólska, Agnieszka Kuc, Adam Pikul, Daria Szewczyk, Paulina Płochocka, Adam Sieradzki

**Affiliations:** ^1^ Department of Experimental Physics Faculty of Fundamental Problems of Technology Wrocław University of Science and Technology Wybrzeże Wyspiańskiego 27 Wrocław 50‐370 Poland; ^2^ Institute of Low Temperature and Structure Research Polish Academy of Sciences Okólna 2 Wrocław 50‐422 Poland; ^3^ Cavendish Laboratory University of Cambridge Cambridge UK; ^4^ Helmholtz‐Zentrum Dresden‐Rossendorf, HZDR Bautzner Landstraße 400 01328 Dresden Germany; ^5^ Center for Advanced System Understanding, CASUS Conrad‐Schiedt‐Straße 20 02826 Görlitz Germany; ^6^ Laboratoire National des Champs Magnétiques Intenses 143 Avenue de Rangueil Toulouse 31400 France

**Keywords:** broadband emissions, crystal structures, phase transitions, self‐trapped excitons, site‐dependent Mn^2+^ emissions

## Abstract

A novel 0D organic–inorganic hybrid manganese bromide, (TMBM)_2_MnBr_4_, based on bromomethyltrimethylammonium (TMBM) cations and isolated [MnBr_4_]^2^⁻ tetrahedra, is synthesized and structurally characterized. The material undergoes two temperature‐induced phase transitions and exhibits intense broadband green photoluminescence at low temperatures. Detailed structural, spectroscopic, and thermodynamic analyses reveal that the emission behavior is governed by the interplay between exciton dynamics and cation ordering. The competition between self‐trapped exciton (STE) formation and energy transfer to Mn^2^⁺ centers enables tunable emission, while variations in Mn─Br bond lengths across crystallographically distinct tetrahedra modulate crystal field strength and emission energy. These insights into structure–property relationships in low‐dimensional manganese halides offer promising avenues for the design of efficient, tunable luminescent materials and multifunctional magneto‐optical devices.

## Graphical abstract

A novel zero‐dimensional hybrid manganese bromide, (TMBM)_2_MnBr_4_, composed of isolated [MnBr_4_]^2^⁻ tetrahedra and TMBM⁺ (bromomethyltrimethylammonium) cations, exhibits two temperature‐driven phase transitions and intense green photoluminescence. Structural and spectroscopic analyses reveal that the emission arises from competition between self‐trapped exciton formation and Mn^2^⁺ energy transfer, modulated by cation ordering and Mn─Br bond variations. These findings highlights its potential for tunable luminescent applications.

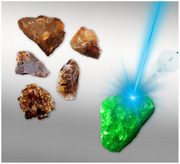

## 1 Introduction

Low‐dimensional (2D, 1D, and 0D) hybrid organic–inorganic perovskite‐like structures represent a promising and rapidly evolving class of materials with broad application potential across various scientific and technological fields.^[^ ^1^, ^2^, ^3^ ^]^ Primarily derived from self‐assembled metal‐organic frameworks with adjustable composition, this class of materials offers unique advantages for light‐emitting applications enabled through structural design. Moreover, the low‐dimensional perovskites are endowed with an unprecedented flexibility concerning their crystal structure, which allows to use of structural distortions to achieve strong, broadband light emission.^[^ ^4^, ^5^ ^]^

Although many reported low‐dimensional perovskites exhibit excellent luminescence properties,^[^ ^6^, ^7^, ^8^, ^9^, ^10^, ^11^ ^]^ the toxicity of Pb^2+^ and their limited stability constrain practical applications, thereby driving the search for lead‐free alternatives.^[^ ^12^, ^13^ ^]^ Lead‐free organic–inorganic metal halides present the advantages of environmental friendliness, stability, and broadband emission. They have emerged as a versatile platform for tuning photoluminescence (PL) owing to their highly efficient and adjustable emission properties, particularly in the case of inorganic [MnBr_4_]^2‐^ ions.^[^ ^14^, ^15^, ^16^, ^17^, ^18^, ^19^, ^20^, ^21^ ^]^ The MnBr_4_‐based compounds were and still are a hotspot for the solid‐state physics community, as they offer great potential for a cheap and simple source of white light. The excellent optical properties of luminescent Mn^2+^ complexes, especially the color‐tunable, high‐efficiency, and long‐lived emission, make them a promising material system to be applied in the optoelectronics field.^[^ ^22^, ^23^, ^24^, ^25^, ^26^, ^27^, ^28^, ^29^, ^30^, ^31^, ^32^, ^33^, ^34^ ^]^ Remarkably, the 0D hybrid MnBr_4_‐based halides have become the basic unit of choice for photo‐excited luminescent materials exhibiting bright photoluminescence,^[^ ^14^, ^17^, ^18^ ^]^ due to their diverse coordination possibilities and adaptable emission characteristics.^[^ ^35^, ^36^, ^37^, ^38^, ^39^ ^]^ The octahedrally coordinated Mn^2+^ ions show broadband red emission, but the tetrahedrally coordinated Mn^2+^ ions exhibit green emission, both of which are attributed to the d‐d transition of Mn^2+^.^[^ ^17^, ^18^, ^40^ ^]^

Much of the controversy in MnBr_4_‐based materials has revolved around the broadband emission. This emission mechanism has been widely explored using diverse experimental techniques.^[^ ^21^, ^25^, ^26^, ^27^, ^28^, ^30^, ^31^, ^32^, ^33^, ^34^, ^35^, ^36^, ^38^, ^40^, ^41^, ^42^, ^43^, ^44^, ^45^ ^]^ Some reports suggest the formation of a localized magnetic polaron in the quantum well of the Mn‐based compound.^[^ ^45^, ^46^ ^]^ The localized excitonic magnetic polaron (EMP) is formed by the sp‐d interaction between the individual spins of carriers in the d‐d transition exciton and the Mn^2+^ spins of the ferromagnetically coupled Mn–Mn.^[^ ^19^, ^47^, ^48^, ^49^ ^]^ The PL energy of the EMP displays a characteristic non‐monotonic temperature dependence resulting mainly from the Coulomb repulsion between d‐d electrons and the crystal field potential.^[^ ^50^ ^]^ However, MnBr_4_‐based compounds, in addition to the characteristic features listed for EMP, also show a large Stokes shift. Therefore, the current consensus is that the efficient red/green emission comes from the radiative recombination of self‐trapped excitons (STEs) or ^4^T_1_ to ^6^A_1_ transitions of Mn^2+^ ions.^[^ ^14^, ^15^, ^41^, ^51^, ^52^, ^53^ ^]^ STEs are excitonic states that become localized due to strong electron‐phonon coupling, typically in low‐dimensional or soft‐lattice materials. Upon excitation, the exciton induces local lattice distortions that create a potential well, effectively trapping the exciton.^[^ ^54^ ^]^ This process often leads to broadband emission with large Stokes shifts and long lifetimes, making STEs highly relevant for luminescent applications in lead‐free and low‐dimensional hybrid materials.^[^ ^5^, ^55^, ^56^, ^57^, ^58^ ^]^

The main goal of this paper is to provide insights into the optical response of the MnBr_4_‐based compounds, particularly the mechanism of the broadband emission at low temperatures. To achieve that, we synthesized a new lead‐free, low‐dimensional compound, and we studied the properties of the compound using several experimental methods (see sec. Materials and Methods). We reveal the complex nature of the emission, which is related to the ordering or statistical freezing of organic cations and STEs at low temperatures. These effects compete with electron‐phonon interactions and determine the temperature‐ and magnetic‐field‐dependent photoluminescence, especially at low temperatures. By performing photoluminescence measurements in a high magnetic field, we probe the behavior of STEs. Our studies provide additional information on the properties of the optical response of MnBr_4_‐based emitters.

## 2 Results and Discussion

### 2.1 Crystal Structure and Phase Behavior

**Figure 1:**
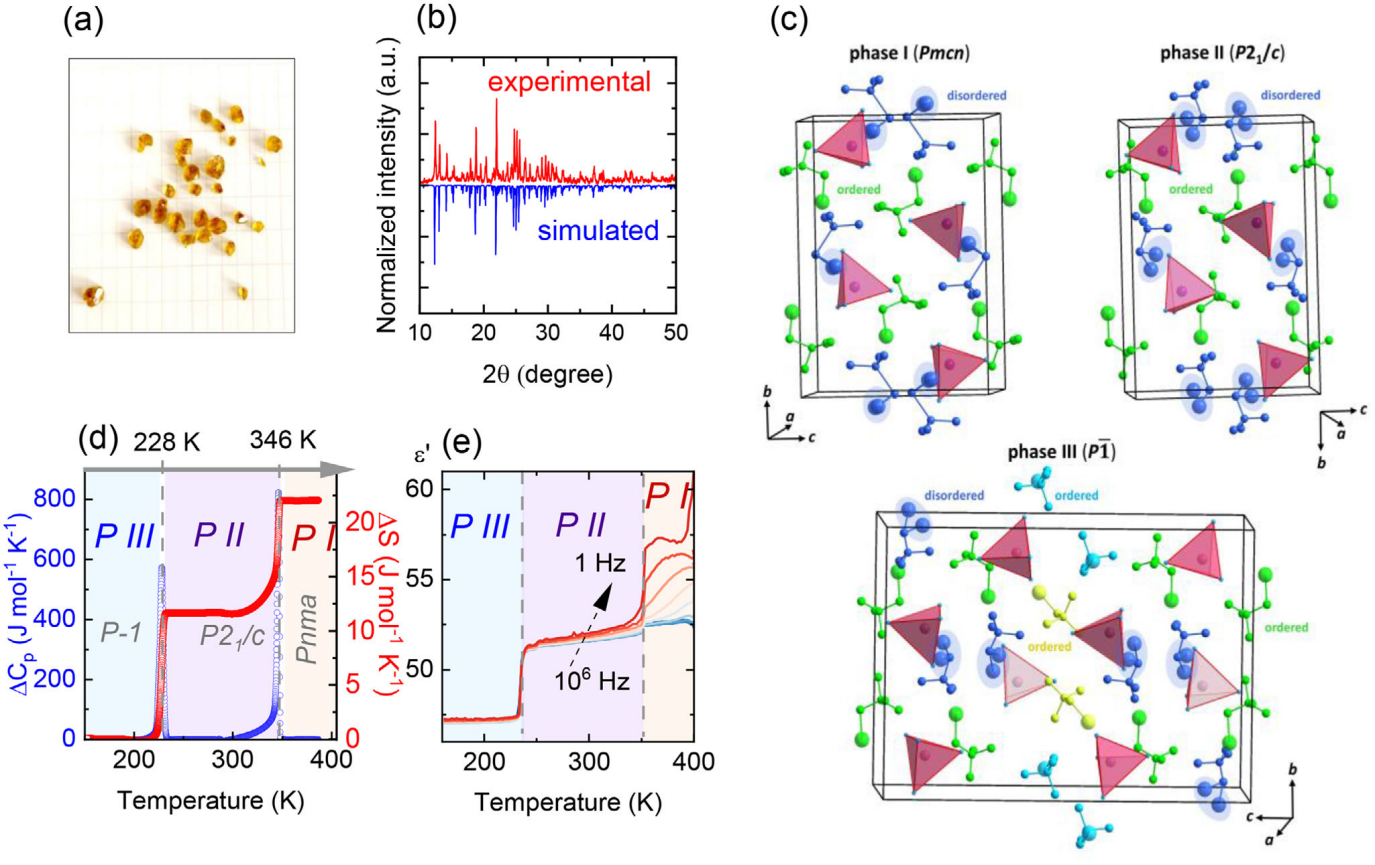
a) Photo of (TMBM)_2_MnBr_4_ crystals. b) Comparison of powder XRD patterns of experimental and simulated data. c) The crystal structure of (TMBM)_2_MnBr_4_ in the HT phase I, intermediate phase II, and LT phase III. d) Changes in heat capacity and entropy related to the phase transitions of (TMBM)_2_MnBr_4._ e) Dielectric permittivity plotted as a function of temperature.

(TMBM)_2_MnBr_4_ crystallizes as amber‐colored single crystals (see **Figure** **1**a), adopting the monoclinic symmetry with *P*2_1_/c space group. The powder XRD pattern of the experimental product is shown in Figure 1b, and it is consistent with the simulated XRD pattern based on our single‐crystal structure data. The crystal structure, depicted in Figure 1c, is characterized by isolated [MnBr_4_]^2‐^ tetrahedra surrounded by TMBM^+^ organic cations. Three crystal polymorphs are observed upon varying temperature: the high‐temperature (HT) phase I is of orthorhombic *Pmcn* symmetry, the intermediate phase II (stable from 350 to 250 K) has *P*2_1_/c symmetry, and finally, the low‐temperature (LT) phase III (below 250 K) adopts triclinic *P* $\bar{1}$ space group (see Table S1, Supporting Information). A characteristic feature common to all phases is the presence of disordered bromine atoms in brominated TMBM^+^ cations. In phases I and II, half of the symmetry‐inequivalent TMBM^+^ cations exhibit positional disorder, whereas in phase III, this disorder is observed in 3 out of 8 TMBM^+^ cations (see Figure 1c; Figure S1, Supporting Information). The positional frustration observed for the brominated molecular species arises from Br···Br interactions between the bromine atoms of the TMBM^+^ cations and the bromide ligands of the [MnBr_4_]^2_^tetrahedra. The phase transition from phase I to phase II is associated with the reorientation of disordered molecules and a change in the configuration of C─Br···Br interactions. In both phases I and II, Br atoms from the ordered molecules are oriented toward the vertices of the MnBr_4_ tetrahedra, forming stable C─Br···Br halogen bonds. However, in phase I, the disordered molecules interact with two neighbouring Mn^2+^ tetrahedra, effectively acting as halogen‐bonded bridges between them. In contrast, in phase II, these molecules reorient toward the edge of a single [MnBr_4_]^2‐^ anion and interact with two bromine ligands from the same tetrahedron. The transition to the low‐temperature phase III involves further reorientation of selected ordered and disordered TMBM^+^ cations and the activation of C─H···Br hydrogen bonds. These interactions contribute to increased distortion of the [MnBr_4_]^2‐^ tetrahedra.

In phases I and II, the lattice parameters reflect the distances between Mn^2+^ ions along specific crystallographic directions. In phase III, a doubling of the c parameter is observed, indicating a loss of translational symmetry along the axis. This structural change is associated with significant reorientation and partial ordering of the molecular substructure, as illustrated in Figure 1c. The shortest Mn^2^⁺···Mn^2^⁺ separations are 8.08, 7.89, and 7.80 Å in phases I, II, and III, respectively. For comparison, the analogous Mn^2^⁺···Mn^2^⁺ distance in (TDMP)₂MnBr₄ (TDMP = trans‐2,5‐dimethylpiperazinium) is 6.8 Å, a value indicative of very weak ferromagnetic interactions and a predominantly paramagnetic behavior.^[^ ^46^ ^]^ This comparison suggests that the compound investigated in this study also exhibits paramagnetism.

The differential scanning calorimetry (DSC) measurements showed the presence of two heat anomalies at T_1_═250 K (228 K) and T_2_═350 K (346 K) during the heating (cooling), which confirms the existence of three crystal phases (see Figure S2, Supporting Information). The structural analogue (TMBM)_2_CdBr_4_ undergoes one phase transition at 314 K from the HT *Pnma* to the LT *P*2_1_ */c* phase.^[^ ^59^ ^]^

The transition entropies, estimated from anomalous heat capacity, are ∆S = 11.8 J mol^−1 ^K^−1^ for the III to II phase and 10.34 J mol^−1 ^K^−1^ for the II to I transition (Figure 1d). These large entropy changes are characteristic of order–disorder transitions involving molecular reorientations. A similar trend is observed in [(C_2_H_5_)_4_N][MnBr_4_], where order‐disorder dynamics and cation rearrangement lead to comparable entropy jumps. These results highlight the central role of organic cation dynamics in the thermally driven transitions of (TMBM)_2_MnBr_4_.^[^ ^60^ ^]^

To probe dynamic processes associated with the observed phase transitions, temperature‐ and frequency‐dependent dielectric permittivity was measured (Figure 1e). The real part (ɛ′) shows distinct step‐like increases at 228 and 346 K, corresponding to the thermally induced phase transitions. Below 228 K (phase III), ɛ′ ≈ 47.5, increasing to ≈52.5 in phase II and further to ≈56 in phase I. The sharp dielectric changes confirm the first‐order character of the transitions. Above 346 K, ɛ′ increases gradually, suggesting the onset of thermally activated conductivity.

IR and Raman spectroscopy, performed from 10 to 400 K (Figure S3, Supporting Information), offers further insights into the phase transitions. Vibrations above 400 cm^−^¹ originate from the internal vibrations of the TMBM⁺ cations, while lower‐frequency bands correspond to the stretching and bending vibrations of [MnBr_4_]^2‐^ tetrahedral units and lattice modes.^[^ ^61^, ^62^, ^63^ ^]^ The tentative assignment at selected temperatures is given in Table S2 (Supporting Information).

To understand the thermal changes observed in the spectra, a simplified factor group analysis can be implemented. According to SCXRD, the primitive cell of the HT phase consists of four formula units, giving 465 optical modes. Among those, 187 (72B_1u_ + 43B_2u_ + 72B_2u_) are IR‐active. In the intermediate phase II, the size of the primitive cell does not change, but the number of optical phonons increases to 231 (116A_u_ + 115B_u_) due to activation of silent A_u_ modes in the monoclinic phase. Further decreasing of symmetry to the triclinic phase III leads to doubling of the primitive cell, resulting in an increase of IR‐active optical phonons to 465A_u_.

**Figure 2:**
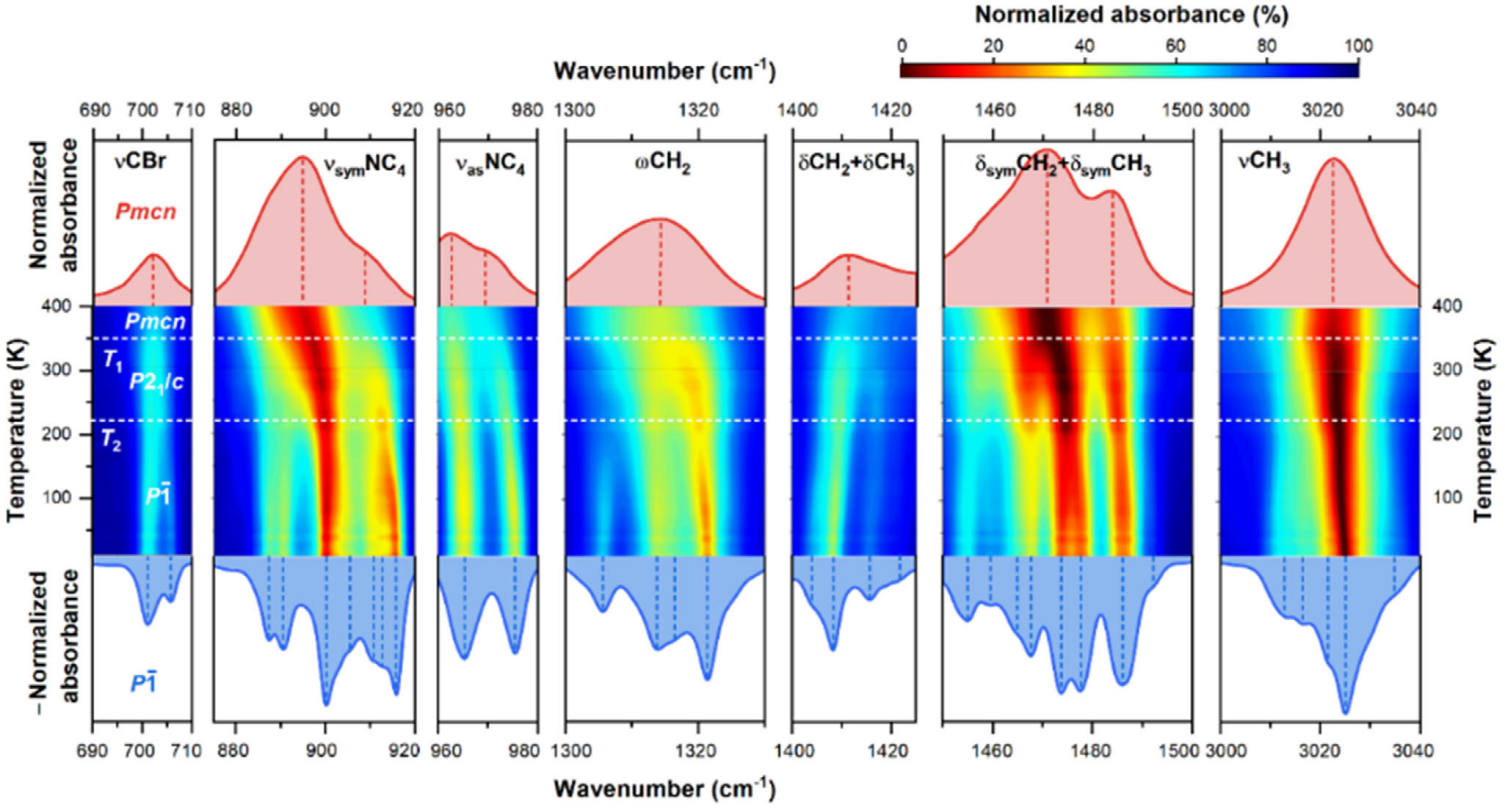
A color map of absorption magnitudes based on a thermal evolution of IR spectra measured for (TMBM)_2_MnBr_4_; white horizontal lines correspond to mean phase transition temperatures obtained from the DSC experiment.

Figure S3 (Supporting Information) shows that the number of observed IR bands in the HT and intermediate phases is nearly the same, in contrast to the significant increase in the LT phase. The pronounced splitting of bands at 350 K can be well visualized using a thermal color map of absorption magnitudes presented in **Figure** **2**. The results of the fitting demonstrate variations in position and bandwidth at phase transition temperatures for several bands, are shown in Figures S4, S5 (Supporting Information). The changes observed at 228 K are much sharper and more pronounced than at 350 K, suggesting that the contribution of organic cations to the mechanism of transformation at 228 K is significant. Since the estimated Δ*S* value is high at 228 K, the structural units building the crystal are not bound by hydrogen bonding, and the jumps in positions and width of bands involving vibrations of methyl and methylene groups are stronger, one may suspect that the disorder also involves the dynamics of H‐atoms, which is supported by higher FWHMs (full at half maximum) of IR bands assigned to CH_2_ group vibrations (Figure S4, Supporting Information). The small changes at 228 K for the stretching vibrations of the NC_4_ cation skeleton indicate the reorientational nature of the changes rather than within the geometry of the TMBM^+^ cations.

The phase transition from low temperature to the intermediate phase (228 K) is related to the change in the ratio between the number of inequivalent ordered and disordered cations from 1.6 to 1 and their interdependent reorientations with respect to tetrahedral inorganic anions. Furthermore, based on changes of calculated structural parameters for [MnBr_4_]^2‐^ tetrahedra, relative to the values for the intermediate phase (RT), i.e., volume (+1.3–2.4%, depending on inequivalent tetrahedron), distortion index (23.3–436.1%), and bond angle variance (from −65.8 to 94.5%),^[^ ^64^ ^]^ it can be concluded that the anionic units are significantly deformed in the LT phase (see details in Table S3, Supporting Information). All these observations provide a reasonable explanation for the high Δ*S* value obtained from thermal measurements at 228 K.

Since the pattern of Br‐atoms disorder of the TMBM^+^ cations during phase transition to the HT phase (350 K) is comparable, this transformation is mainly driven by reorientations of the TMBM^+^ cations and followed by deformation of inorganic units with regard to the RT phase. With the decrease in volume with a magnitude of −5.6%, the distortion index at 355 K is 4.6 times higher, and the bond angle variance is 2.5 times higher in comparison to the intermediate RT phase (Table S3, Supporting Information).

Apart from the two expected anomalies at 350 and 228 K, the third anomaly near 40 K (T_3_) can be noticed in Figure 2 as a change in the width and intensity of the entire complex contours. The data presented in Figures S4, S5 (Supporting Information) barely show this anomaly. It can be visible as unusual and weak softening of IR bands at very low temperatures upon cooling, demonstrating, this is rather connected with subtle effects. However, the anomaly at 40 K is observed for most of the bands, suggesting that the positions or dynamics of organic cations are affected (Figure 2; Figure S6, Supporting Information). Since the crystal structure is still disordered at 120 K, as evidenced by X‐ray diffraction studies, one could expect the subsequent ordering at certain temperatures upon cooling. A slight increase in the intensity of the bands and an anomaly in the course of some FWHMs suggest that already at a temperature of ≈110–120 K, ordering or simply statistical freezing and a significant slowdown in the dynamics of the cations may begin. This observation naturally leads to a discussion on magnetic ordering in the crystal.

To improve our understanding of the low‐temperature behavior in the (TMBM)_2_MnBr_4_ crystal, we measured magnetic and thermal properties down to liquid ^4^He temperatures to check if the sample exhibits phase transitions below 150 K. The specific heat measurement revealed a very blurred and relatively weak inflection around 40 K, which corresponds to the same temperature as the observed additional anomaly in the IR spectra (see Figure S7, Supporting Information).

To explore possible low‐temperature ordering and magnetic coupling, we performed magnetic measurements down to 2 K. Magnetic susceptibility data of (TMBM)_2_MnBr_4_ are demonstrated in Figure S8 (Supporting Information). The inverse magnetic susceptibility, χ^−1^(T), of the compound exhibits a linear temperature dependence across the entire measured range, yielding an effective magnetic moment of *µ*_eff_ ═ 5.90(1) µ_B_ and the paramagnetic Curie‐Weiss temperature *θ*_p_═0.3(2) K. The value of *µ*_eff_ is nearly the same as the theoretical value *µ*_eff_ ═ 5.92 *µ*_B_ calculated for free Mn^2+^ ions within the Russell‐Saunders coupling (*S* = 5/2, *L* = 0, and *J* = 5/2). The value of *θ*_p_ indicated the presence of very weak (if any) ferromagnetic interactions between the manganese magnetic moments. Indeed, the compound remains paramagnetic at least down to 2 K, which is confirmed by the behavior of the field dependence of magnetization M(H), showing paramagnetic saturation (Brillouin‐like curve) with the saturated magnetic moment of ≈5.01(1) *µ*_B,_ which is again very close to the theoretical value 5 *µ*_B_ for Mn^2+^ ions (see Figure S8c, Supporting Information). In case of (TMBM)_2_MnBr_4_, the shortest Mn–Mn distance is 7.76 Å. Ad mentioned above, ferromagnetic interactions are hardly visible in the compound studied, while some very weak ferromagnetic interactions were reported for (TDMP)MnBr_4_ crystal with the shortest Mn–Mn distance of 6.8 Å,^[^ ^46^ ^]^ which suggests that in the (TMBM)₂MnBr₄ sample, the Mn–Mn distance is too long for ferromagnetic interactions to occur.

### 2.2 STE Behavior and Site‐Dependent Mn^2^⁺ Emission

To provide a deeper understanding of the behavior of the studied material at low temperatures, a comprehensive series of optical measurements was performed. To determine the optical bandgap of (TMBM)_2_MnBr_4_, UV–vis diffuse reflectance spectrum was recorded. Figure S9 (Supporting Information) shows the absorbance as a function of energy fitted by Tauc's Equation:

(1) $${\left(\alpha hv\right)}^{\frac{1}{n}}=A\left(hv-E_{g}\right)$$

(n═2 for indirect bandgap n═1/2 for direct bandgap).^[^ ^65^ ^]^ The estimated bandgap is equal to 4.67 eV. Usually, for MnBr_4_‐based compounds, the bandgap ranges between 2.6 and 4.7 eV.^[^ ^20^, ^23^, ^37^, ^51^ ^]^ The electronic band structure of (TMBM)_2_MnBr_4_ has been calculated using the DFT method (see Figure S10, Supporting Information). The compound (TMBM)_2_MnBr_4_ exhibits flat band edges, suggesting the presence of highly localized electronic states, which aligns with the typical electronic structure of 0D organic–inorganic hybrid metal halides.^[^ ^22^, ^34^, ^66^ ^]^ Moreover, the flatness of both the valence band maximum (VBM) and the conduction band minimum (CBM) indicates minimal intermolecular coupling within the [MnBr_4_]^2−^ tetrahedra, confirming that each tetrahedron acts as an independent emission center^[^ ^23^ ^]^ – a crucial factor in achieving highly efficient luminescence.^[^ ^34^, ^67^ ^]^

**Figure 3:**
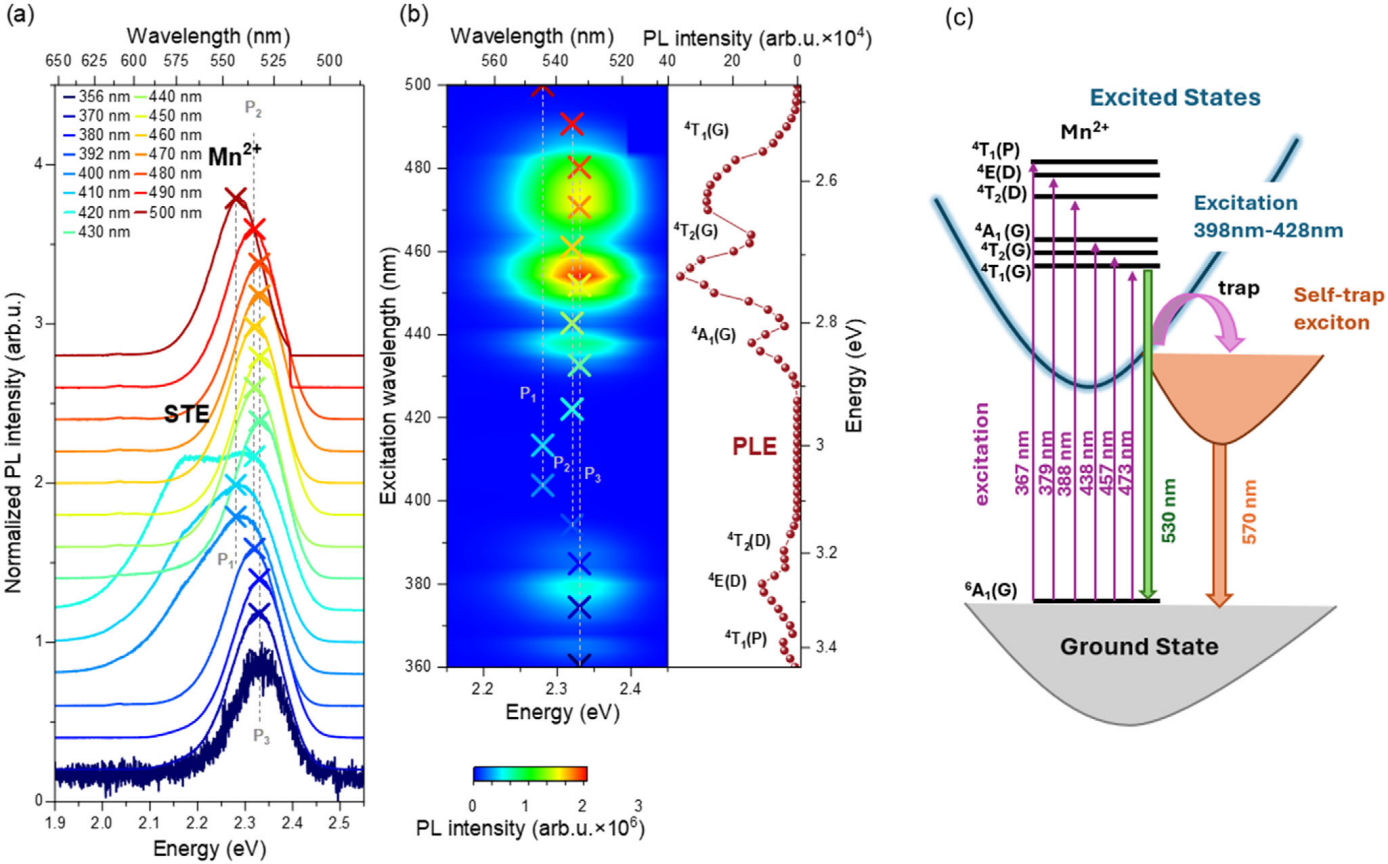
a) A comparison of normalized PL spectra measured at 4 K for (TMBM)_2_MnBr_4_ single crystal at different excitation wavelengths; the vertical lines P_1_, P_2_, and P_3_ correspond to different positions of Mn^2+^ in the crystal structure; STE and Mn^2+^ indicate bands corresponding to exciton and manganese(II) emission. b) A color map of PL spectra measured as a function of excitation wavelength at 4 K together with PLE spectrum; the vertical lines P_1_, P_2_, and P_3_ correspond to different positions of Mn^2+^ in the crystal structure; points correspond to the positions of the maxima of the peaks from PL spectra measured at selected excitations in (a); the colors of the points in Figure 3b correspond to the colors in the Figure 3a. c) Schematic illustration of the energy transition and exciton dynamics leading to the dual‐band emission in (TMBM)_2_MnBr_4._

Building on this foundation, it was essential to continue examining the optical properties of (TMBM)_2_MnBr_4_. We performed photoluminescence excitation (PLE) and reflectivity (R) measurements at 4 K to gain insight into the PL processes‐ see Figure S11 (Supporting Information). We observed a large Stokes shift between absorption (PLE) and emission (PL), which takes the value of 290 meV. We then performed PL measurements at 4 K as a function of excitation wavelength in the 356–500 nm. The results are presented in **Figures** **3**a and S12 (Supporting Information). The PL spectra present broadband d‐d emission of tetrahedrally coordinated Mn^2+^ ions with a maximum near 2.30 eV (539 nm).^[^ ^68^ ^]^ The PLE spectrum of (TMBM)_2_MnBr_4_, monitored at 530 nm, consists of six bands originating from transitions between ground state ^6^A_1_ and excited states of ^4^T_1_(G) (2.62 eV, 473 nm), ^4^T_2_(G) (2.71 eV, 457 nm), and ^4^A_1_(G) (2.83 eV, 438 nm), and the second triplet of ^4^T_2_(D) (3.20 eV, 388 nm), ^4^E(D) (3.27 eV, 379 nm), and ^4^T_1_(P) (3.38 eV, 367 nm),^[^ ^41^, ^46^, ^51^, ^52^ ^]^ see Figure 3b.

Similar green emission with maximum between ca. 505 and 530 nm (2.34–2.46 eV) was observed for Mn^2+^‐doped inorganic zinc bromides and between 504 and 520 nm (2.38–2.46 eV) for hybrid inorganic–organic manganese(II) tetrahalides composed of varied ammonium and phosphonium organic cations.^[^ ^42^, ^69^ ^]^ Previous studies of hybrids composed of [MnBr_4_]^2‐^ tetrahedra, showed that the efficiency of Mn^2+^ green emission is directly related to the Mn–Mn distances, depending on the size of the organic cations.^[^ ^69^ ^]^ In (TMBM)_2_MnBr_4_, the closest distance between ions Mn^2+^ is 7.76 Å, which is a rather low value that allows for non‐radiative energy transfers between Mn^2+^ centres. The transfer can influence the precise tuning of the emission wavelength.

A color map of all measured PL spectra, displayed in Figure 3b, shows a clear correlation with the excitation spectrum. As expected, the strongest PL signal was obtained when the excitation energy was resonant with ^4^T_1_(G) and ^4^T_2_(G) transitions. Furthermore, the relative intensity of PL peaks agrees well with the intensity of excitation bands; therefore, to efficiently excite Mn^2+^ levels the best excitation energy ranges between 445 and 475 nm. It is worth noting that the position of the PL peak assigned to the Mn^2+^ ion emission, observed around 2.30 eV (539 nm), fluctuates between three positions, i.e., P_1_ at 2.29 eV (542 nm), P_2_ at 2.32 eV (535 nm), and P_3_ at 2.33 eV (531 nm). This effect is even more visible in Figure 3a, comparing the shapes of normalized spectra at selected excitation wavelengths. The reason for maximum variation is the low symmetry of the phase III, which is related to the presence of crystallographically unequal sites of manganese ions in the structure. The variety of Mn─Br bond lengths in different tetrahedra changes crystal field (CF) strength, determining the energy of emission.^[^ ^42^ ^]^; the average Mn─Br lengths for [Mn2Br_4_]^2‐^ (2.5012 Å) + [Mn4Br_4_]^2‐^ (2.5008 Å), [Mn3Br_4_]^2‐^ (2.4962 Å), and [Mn1Br_4_]^2‐^ (2.4934 Å) tetrahedra made it possible to assign them to P_1_, P_2_, and P_3_ positions, respectively. The lowest CF representing P_1_ dominates the PL spectrum when the excitation energy does not match the absorption peaks (see Figure 3b). The P_2_ and P_3_ positions, corresponding to stronger CF, have a strong contribution in the PL spectra, when the excitation energy is slightly red‐ or blue‐shifted with regard to the peak maximum, respectively.

Another interesting feature observed in Figure 3a and Figure S11 (Supporting Information) is the appearance of an additional weak band near 2.17 eV (570 nm), when the excitation energy ranges between 398 and 428 nm, i.e., in the range in which Mn^2+^ ions are weakly excited. This emission is relatively broad; therefore, it was attributed to the self‐trapped exciton (STE) emission related to defects. Figure 3c illustrates a schematic configurational coordinate diagram with the process of exciton self‐trapping. A similar dual band emission STE (510 nm) and Mn^2+^ (605 nm) was observed for 1D DMEDAPb_x_Mn_1‐x_Br_4_ (DMEDA═*N*,*N*′‐dimethylethylenediammonium) with Mn^2+^ in octahedral coordination.^[^ ^70^ ^]^ Another dual‐band PL from polaronic and bipolaronic excitons (STE) was observed for 0D C_4_H_14_N_2_MnBr_4_ at 516 and 623 nm.^[^ ^45^ ^]^

To understand the origin of the emission of both bands, the decay times were recorded at 16 K using 407 nm excitation and monitored at 540 nm in the µs range and at 560 nm in the ns range (see Figure S13, Supporting Information). Generally, for compound based on [MnBr_4_]^2‐^ the average PL lifetime for d‐d transition is expected to be in the range of ms.^[^ ^15^ ^]^ The fitting was done using a two‐phase exponential decay function, because of more than one positions of Mn^2+^ ions. For the same reason, we calculated the average decay time of 0.34 µs. This value is relatively shorter in comparison to similar 0D hybrid compounds with tetrabromomanganate(II) ions, i.e., 3.6 µs for C_4_H_14_N_2_MnBr_4_^[^ ^45^ ^]^ and 19–620 µs (RT) for a number of [cat]_2_MnBr_4_ (cat═organic cation) compounds compiled by Mao et al.^[^ ^44^ ^]^ and Morad et al.^[^ ^42^ ^]^ For compounds with mixed cations, [(TEA)(TMA)]MnCl_4_ and [(TPA)(TMA)_3_](MnCl4)_2_ (TEA═tetraethylammonium, TMA═tetramethylammonium, and TPA═tetrapropylammonium) the estimated decay times were much longer, in the order of a few milliseconds (3.74 and 3.63 ms respectively).^[^ ^43^ ^]^

The STE time decay response was fitted using a monoexponential decay; the resulting time was 3.50 ns. These orders of magnitude and rapid vanishing with increasing temperature (Figure S14, Supporting Information) are determinants confirming that the band is originated from STE.^[^ ^70^ ^]^

**Figure 4:**
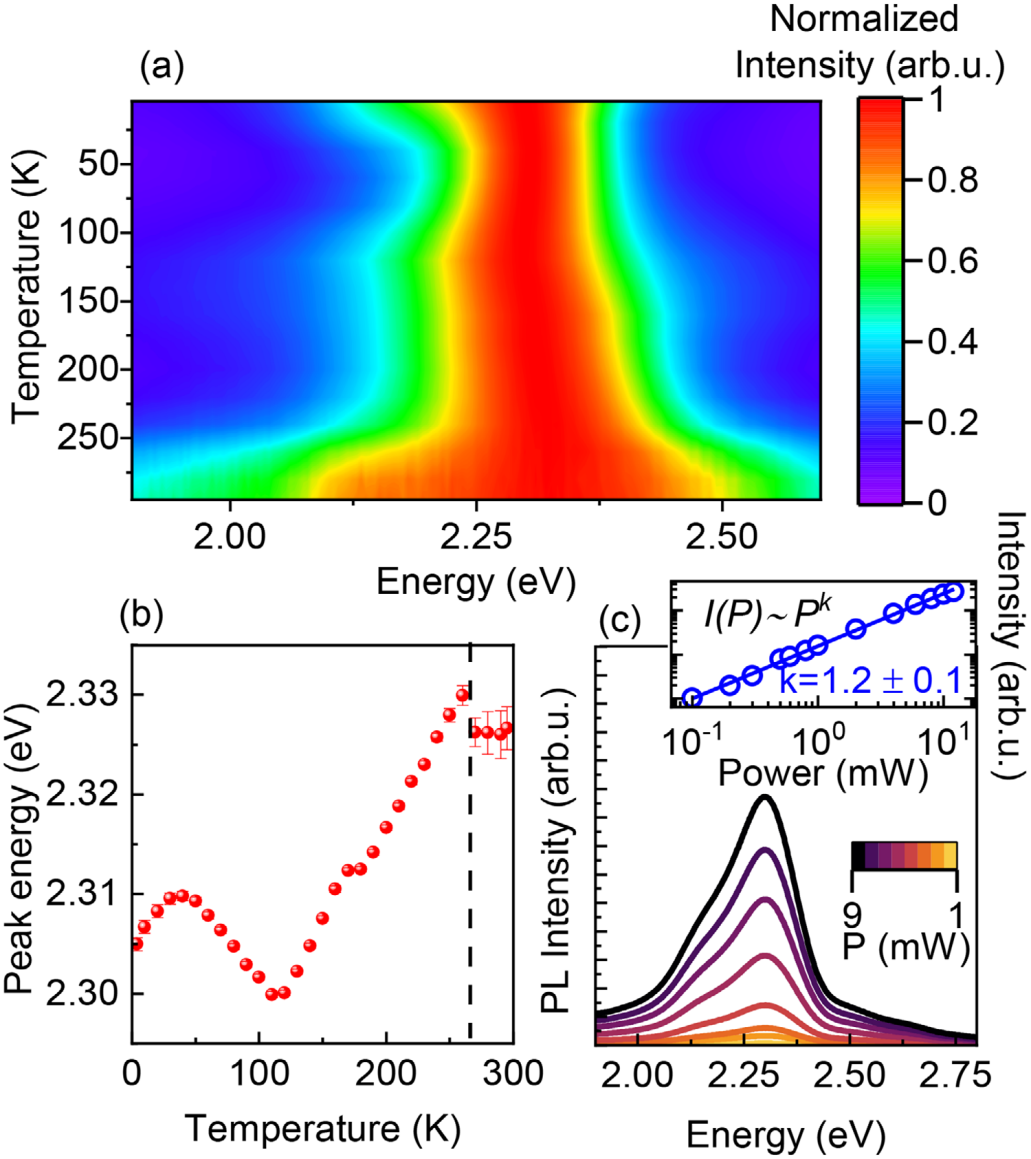
a) Normalized PL intensity spectra of (TMBM)_2_MnBr_4_ at different temperatures; b) Temperature dependence of the integrated PL peak intensity, determined by the area under the fitted spectral curves; c) Power‐dependence of PL at 4 K using the excitation wavelength of 407 nm; inset in panel (c) shows log‐log plot of the PL‐integrated intensity versus laser power.

To better understand the mechanism of the dual band emission in the studied compound, we conducted PL measurements as a function of temperature (4–295 K) measured at 407 nm excitation (**Figure** **4**a,b; Figure S14, Supporting Information). Since the formation of STEs is a thermally activated process, it is expected that temperature will influence the relative PL energy and intensity of (TMBM)_2_MnBr_4_. By closely analysing the change in the peak position as a function of the temperature, we find a clear redshift below 250 K upon cooling, owing to the structural phase transition between phases II and III. This behavior was observed in most Mn‐based phosphors and was attributed to the increasing crystal‐field strength following the lattice shrinkage.^[^ ^19^, ^46^, ^71^, ^72^ ^]^ At ≈110–120 K, the curve reverses, and a slight blueshift occurs to about 40 K. Upon further cooling to 4.2 K, the PL peak exhibits another minor redshift. This behavior of PL as a function of temperature raises additional questions about the origin of emission in low‐dimensional perovskite‐like compounds. It is clear that below 110–120 K the nature of the emission is more complex than the d‐d transition in tetrahedrally coordinated Mn^2+^ ions, and this behavior may indicate some additional paths of energy transfer or Mn–Mn interactions.^[^ ^19^ ^]^ Such characteristic thermal variations have already been reported.^[^ ^49^, ^50^ ^]^ and attributed to the formation of a polaron or light‐induced.^[^ ^73^ ^]^ weak magnetic ordering between Mn–Mn ions.^[^ ^19^ ^]^ However, due to the lack of observed magnetic ordering, the results presented here may support the hypothesis that the anomaly below 40 K may originate from the TMBM^+^ cation ordering or freezing. The band assigned to STE is present at 4 K, however, its intensity quickly drops upon heating and disappears at 50 K, as shown in Figure S15b (Supporting Information). To further confirm the STE process, we fit the activation energy using the Arrhenius formula: $I\left(t\right)=\frac{I_{0}}{Aexp(-\frac{E_{a}}{k_{BT}})}$, where k_B_ is the Boltzmann constant, A is an applicable constant, and I(T) is the PL intensity at temperature (T) (see Figure S15c, Supporting Information). The activation energy determined for Mn^2^⁺ emission (above 50 K) is 25.9 meV. This value is comparable to the activation energies reported for other MnBr₄‐based compounds, such as (TBA)₂MnBr₄ (39 meV) ^[^ ^34^ ^]^ and (TMPEA)₂MnBr₄ (27 meV) ^[^ ^74^ ^]^ Upon UV exposure, the electrons in the ground states are promoted to excited states within Mn^2+^ d–d orbitals. Structural reorganization or localized excitons in the excited states can lead to the formation of STEs, which in turn produce highly efficient broadband photoluminescence with a large Stokes shift as the excitons relax to the ground state. As the temperature increases, the STEs gain enough energy to escape from their trap states, causing enhanced thermal quenching. Consequently, the excitons become more likely to be captured by the Mn^2+ 4^T_1_(G) energy level, resulting in more efficient emission at 2.3 eV.

We performed measurements of PL as a function of the excitation power at 4 K. Figure 4c shows that the PL intensity, presented in the log‐log graph, changes linearly with laser power, excluding emission from permanent defects due to their limiting concentration and saturation of Mn^2+^ emission at high excitation power. The linear power dependence of the emission intensity and the exponent equal to 1.2(1) are signatures of STE emission. To verify and clarify this assumption, a thorough structural analysis should be carried out.

**Figure 5:**
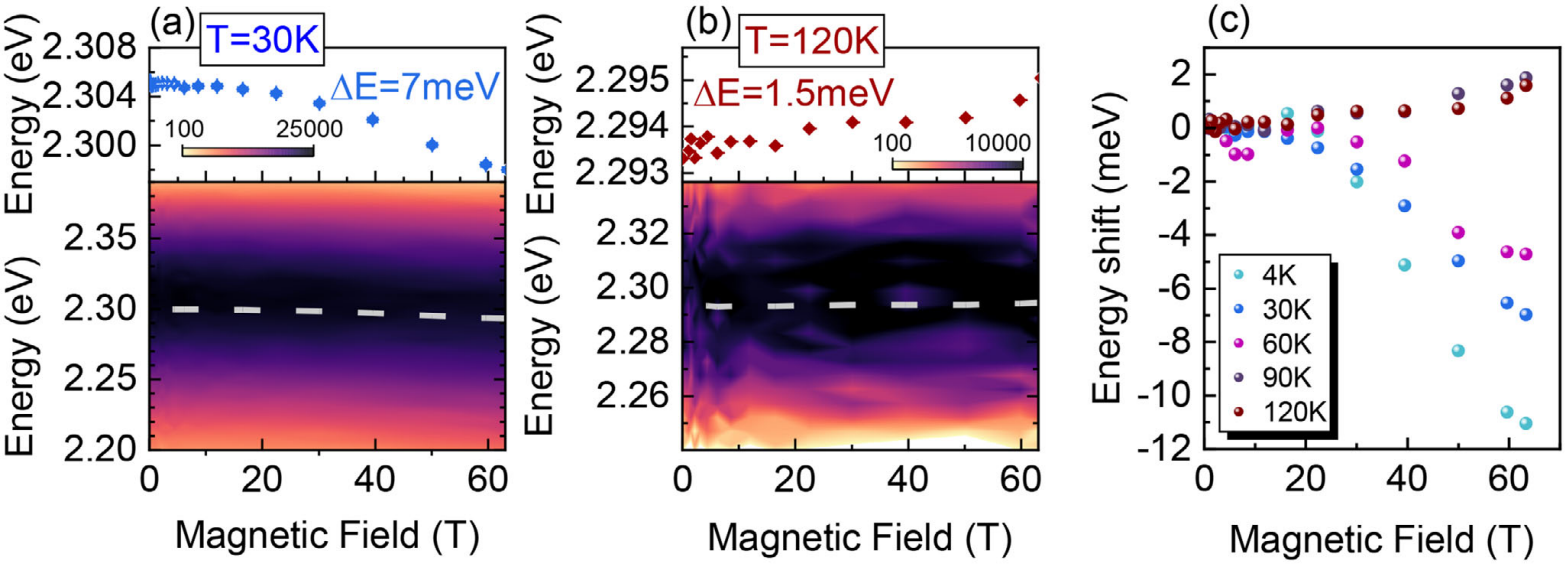
The false‐color plot of the PL spectrum shows the energy shift in extreme magnetic fields (bottom graph) and magnetic field‐induced energy shift of the PL (top graph) a) at 30 K, b) and 120 K. c) Peak energy shift in the magnetic field at 4, 30, 60, 90, and 120 K.

The PL spectra were recorded in an external magnetic field. The (TMBM)_2_MnBr_4_ compound is paramagnetic, which means that a high magnetic field may be required to turn the Mn–Mn spin order. As shown in Figure 5a, in an applied pulsed magnetic field of up to 65 T, the PL peak shows a slight redshift at 30 K. It could be related to the modulation of the Mn–Mn ordering from paramagnetic to partial ferromagnetic ordering.^[^ ^19^, ^49^ ^]^ which is absent in the paramagnetic state at 120 K (see Figure 5b).^[^ ^19^ ^]^ However, it is worth noting that for another low‐dimensional compound, aminoguanidinium lead iodide (AGAPbI_3_), the authors attributed the shift in high magnetic fields at 4 K to STEs behavior.^[^ ^75^ ^]^ Since we also observe the emission of STE, this explanation also seems reasonable for (TMBM)_2_MnBr_4_. The magnetic‐field dependent peak shifts at 4, 30, 60, 90, and 120 K are shown in Figure 5c and Figure S16 (Supporting Information). The field‐induced red shift increases with decreasing temperature, which is consistent with an interplay between the ordering of spins imposed by the external magnetic field and thermal effects. At low temperatures, the localized excitons or the excited states indeed form STE states, which consequently generate a highly efficient broadband PL with redshift under a high magnetic field. As the temperature rises to 60 K, the STEs gain sufficient energy to escape from their trap states, leading to enhanced quenching of STEs. Subsequently, the excitons become more likely to be captured by the Mn^2+ 4^T_1_(G) energy level, resulting in slight blue shift under a high magnetic field.

## 3 Conclusion

This work presents a detailed study of the structural and optical behavior of the lead‐free hybrid manganese bromide (TMBM)_2_MnBr_4,_ which undergoes two distinct phase transitions. Infrared spectroscopy revealed significant structural reorganization at 350 K and more subtle distortions at 228 K, both affecting the local symmetry and cation dynamics. Below 120 K, signs of an additional low‐temperature phase was observed, correlating with the emergence of self‐trapped exciton (STE) emission. Photoluminescence studied show that emission properties are governed not only by Mn^2+^ d‐d transitions but also by STE formation, electron‐phonon coupling, and short‐range magnetic interactions. The strong excitation‐wavelength‐dependent emission under laser irradiation highlights the material/s nonlinear optical response. These findings underscore the interplay between structure, dynamics, and emission mechanisms in low‐dimensional Mn‐based halides, offering insights for the design of next‐generation lead‐free optoelectronic materials.

## 4 Experimental Section

### Sample Synthesis Details

To synthesize the (TMBM)_2_MnBr_4_ crystals, bromomethyltrimethylammonium bromide (Me_3_NCH_2_Br)Br, (TMBM)Br was produced in the first stage using the procedure described previously.^[^ ^76^, ^77^ ^]^ In this manner, 270 µL of trimethylamine (TMA) solution (25 wt.% in H_2_O, Sigma–Aldrich), equivalent to ≈1 mmol of TMA, was mixed with 70 µL of dibromomethane (99%, Sigma–Aldrich) and 1 mL of acetonitrile. The stirring was continued on a magnetic stirrer, and the yellowish precipitate of (TMBM)Br was collected after an hour and air‐dried. Then, 5 mmol (1.1647 g) of dry (TMBM)Br and 2.5 mmol of MnBr_2_ (0.5369 g) were dissolved in 10 mL of water each. MnBr_2_ solution was progressively added to (TMBM)Br solution drop by drop while stirring continuously on a magnetic stirrer. After 1 h, 1 mL of concentrated hydrobromic acid (48 wt.% in H_2_O, Sigma–Aldrich) was added. The solution was left to crystallize slowly. Amber‐colored (TMBM)_2_MnBr_4_ crystals were collected and air‐dried after several days.

### Crystal Structure Investigations

Single‐crystal x‐ray diffraction (SCXRD) measurements were performed using MoKα radiation on an Xcalibur four‐circle diffractometer (Oxford Diffraction) equipped with an Atlas CCD detector and graphite‐monochromated MoKα radiation. Absorption corrections were applied using multi‐scan methods with spherical harmonics, implemented via the SCALE3 ABSPACK scaling algorithm. The crystal structure was solved in Olex2 1.5.^[^ ^78^ ^]^ using SHELXT‐2014/4 and refined with SHELXT‐2018/3.^[^ ^79^ ^]^ The unit cell of phase I (orthorhombic, *Pmcn*) was chosen in the nonstandard setting of the *Pnma* space group in order to maintain the crystallographic directions from phase II. Both phases II (monoclinic, *P*2_1_ */c*) and III (triclinic, *P* $\bar{1}$) were treated as a two‐domain twin, and the H atom parameters were constrained. Experimental details are presented in Table S1 (Supporting Information).

### Thermal Properties

Heat‐flow thermograms were recorded using a high‐resolution Mettler Toledo DSC‐1 calorimeter with a sensitivity of 0.04 µW. Nitrogen served as the purge gas during measurements, which were conducted over a temperature range of 150–390 K with heating and cooling rates of 5 K min^−1^. The excess heat capacity related to the phase transition was determined by subtracting a baseline representing the variation in the absence of the transition. Additionally, the heat capacity at constant pressure for (TMBM)_2_MnBr_4_ was measured between 1.8 and 100 K using the thermal relaxation method in the heat capacity option of the Physical Property Measurements System (PPMS).

### Dielectric Properties

The dielectric properties of a single crystal were measured as a function of frequency and temperature using a broadband impedance analyzer (Novocontrol Alpha). The sample was examined isothermally over a frequency range from 1 Hz to 1 MHz. Measurements were performed in 1 K increments across the temperature range of 150–430 K.

### Magnetic Properties

Magnetic properties of a large collection of randomly oriented single crystals of (TMBM)_2_MnBr_4_ were investigated using a commercial Quantum Design SQUID magnetometer, over the temperature range from room temperature (RT) down to 2 K, and under an external magnetic field up to 70 kOe. The contribution from the weakly diamagnetic sample holder (not shown) was negligible compared to the total measured signal, so no background subtraction was performed.

### Vibrational Spectroscopy Measurements

The RT Raman spectrum (3500–50 cm^−1^) of (TMBM)_2_MnBr_4_ was measured using an FT‐MultiRam spectrometer (Bruker) equipped with a YAG:Nd laser excitation at 1064 nm. The temperature‐dependent mid‐IR spectra (3500–600 cm^−1^), in the 10–320 K range, were collected using a Nicolet iS50 infrared spectrometer (Thermo Scientific) combined with a CS202AE‐DMX‐1AL closed‐cycle helium cryostat (Advanced Research Systems) equipped with thallium bromoiodide (KRS‐5) windows. The mid‐IR spectra above room temperature (300‐400 K) were registered in the 3500–600 cm^−1^ spectral range using a Nicolet iN10 stand‐alone IR microscope (Thermo Scientific) combined with an FTIR600 stage (Linkam) equipped with ZnSe windows. All IR measurements were performed on a KBr pellet; the spectral resolution of all Raman and IR measurements was set to 2 cm^−1^.

### Optical Spectroscopy Setup

The photoluminescence excitation (PLE), photoluminescence (PL), and reflectivity experimental results have been obtained at cryogenic temperatures of ∼4 K by cooling down the sample mounted on the cold finger of a He flow cryostat. A femtosecond‐pulsed Ti‐sapphire laser was used to pump an optical parametric oscillator, which was used as the excitation source to achieve wavelength tuning in a wide range. A 50x microscope objective (Mitutoyo Inc.) with a numerical aperture of 0.55 focused the incident laser or white‐light beam onto the investigated crystal. A UV lamp was used to provide broadband white light.

The decay times at 16 K were measured using an Edinburgh Instruments FLS1000‐DD‐STM spectrofluorometer equipped with an EPL405 picosecond pulsed diode with a centre wavelength of 402.1 nm. The temperature of monocrystals was controlled using a closed‐cycle helium cryostat CS‐204‐FMX‐1SS (Advanced Research Systems) equipped with a cold head DE‐204S

Magneto‐photoluminescence spectra were measured in a nitrogen‐cooled pulsed magnet providing a maximum field of B ≈65 T with a pulse duration of ≈500 ms. The sample was excited with a 407 nm source. The measurements were performed in the Faraday configuration. The sample was installed in a He cryostat in the centre of the magnetic field. PL measurements were performed between 4 and 300 K by using a 407 nm laser light.

### Methodology of Density Functional Theory

The structure of (TMBM)_2_MnBr_4_ was fully optimized (atomic positions and lattice vectors) using AMS 2021 software. The geometry optimization and electronic properties were obtained by employing PBE exchange–correlation functional.^[^ ^80^ ^]^ with the D3(BJ) dispersion correction.^[^ ^80^, ^81^ ^]^ the valence triple‐zeta polarized (TZP) basis sets composed of Slater‐type and numerical orbitals, and scalar zero‐order regular approximation (ZORA).^[^ ^82^ ^]^ Spin‐polarisation was included in all simulations. Normal and Good numerical qualities were used for relaxation and electronic structure simulations, respectively, corresponding to 3x1x1 k‐point sampling. The optimized lattice parameters are *a* = 9.228 Å, *b* = 17.517 Å, *c* = 24.802 Å, *α* = 89.2°, *β* = 90.0°, *γ* = 88.9°, which is in good agreement with experimental data *a* = 9.273 Å, *b* = 17.531 Å, *c* = 24.877 Å, *α* = 89.8°, *β* = 89.4°, *γ* = 89.2°.

## Conflict of Interest

The authors declare no conflict of interest.

## Supporting information



Supporting Information

## Data Availability

The data that support the findings of this study are openly available in zenodo at https://doi.org/10.5281/zenodo.15737436, reference number 15737436.
