# Supplementary material for: Tunable Broadband Emission via Self‐Trapped Excitons and Mn2+ Energy Transfer in a 0D Hybrid Manganese Bromide
Source: Small. 2025 Jul 26;21(38):e04786. doi: 10.1002/smll.202504786 (PMC12462603; doi:10.1002/smll.202504786)
Supplement: Supplementary file 1 — Supporting Information [file SMLL-21-e04786-s001.docx]

**Tunable Broadband Emission via Self-Trapped Excitons and Mn^2+^ Energy Transfer in a Zero-Dimensional Hybrid Manganese Bromide**

*Paulina Peksa ^1 *^, Maciej Ptak ^2^, Mateusz Dyksik ^1^, Alessandro Surrente ^1^, Michał Baranowski ^1^, Dawid Drozdowski ^2^, Anna Gągor ^2^, Julia Osmólska ^2,3^, Agnieszka Kuc ^4,5^, Adam Pikul ^2^, Daria Szewczyk ^2^, Paulina Płochocka ^1,6^ and Adam Sieradzki ^1*^*

1. Department of Experimental Physics, Faculty of Fundamental Problems of Technology, Wrocław University of Science and Technology, Wybrzeże Wyspiańskiego 27, 50-370 Wrocław, Poland
2. Institute of Low Temperature and Structure Research, Polish Academy of Sciences, Okólna 2, 50-422 Wrocław, Poland
3. Cavendish Laboratory, University of Cambridge, Cambridge, UK
4. Helmholtz-Zentrum Dresden-Rossendorf, HZDR, Bautzner Landstraße 400, 01328 Dresden, Germany
5. Center for Advanced System Understanding, CASUS, Conrad-Schiedt-Straße 20, 02826 Görlitz, Germany
6. Laboratoire National des Champs Magnétiques Intenses, 143 Avenue de Rangueil 31400 Toulouse, France
   ^*^ [paulina.peksa@pwr.edu.pl](mailto:paulina.peksa@pwr.edu.pl), [adam.sieradzki@pwr.edu.pl](mailto:adam.sieradzki@pwr.edu.pl)

**Table S1.** SCXRD experimental and refinement data of (TMBM)_2_MnBr_4_ (Mr = 680.87).

| Crystal data | | | |
| --- | --- | --- | --- |
| Crystal system, space or superspace group | Orthorhombic, *Pmcn* | Monoclinic, *P*2_1_/*c* | Triclinic, *P*$\overline{1}$ |
| Temperature (K) | 355 | 293 | 120 |
| *a*, *b*, *c* (Å) | 9.455 (2), 17.542 (4), 12.673 (2) | 9.515 (3), 17.525 (6), 12.543 (4) | 9.273 (3), 17.531 (6), 24.876 (7) |
| *α, β, γ* (°) | 90, 90, 90 | 90, 93.10 (3), 90 | 89.80 (3), 89.41 (3), 89.23 (3) |
| *V* (Å^3^) | 2102.1 (7) | 2088.6 (12) | 4043 (2) |
| *Z* | 4 | 4 | 8 |
| *μ* (mm^-1^) | 12.02 | 12.10 | 12.50 |
| Crystal size (mm) | 0.22 × 0.2 × 0.12 | 0.22 × 0.2 × 0.12 | 0.22 × 0.2 × 0.12 |
|  | | | |
| Data collection | | | |
| *T*_min_, *T*_max_ | 0.297, 1.000 | 0.647, 1.000 | 0.493, 1.000 |
| No. of measured, independent and observed reflections | 7806, 2275, 510 | 7112, 7112, 2944 | 86523, 16120, 10061 |
| *R*_int_ | 0.190 | - | - |
| (sin *θ*/λ)_max_ (Å^-1^) | 0.625 | 0.610 | 0.610 |
|  | | | |
| Refinement | | | |
| *R*[*F*^2^ > 2*σ*(*F*^2^)], *wR*(*F*^2^), *S* | 0.197, 0.567, 1.20 | 0.076, 0.233, 0.89 | 0.089, 0.257, 1.02 |
| No. of reflections | 2275 | 7112 | 16120 |
| No. of parameters | 97 | 104 | 410 |
| No. of restraints | 90 | 7 | 2 |
| Δ*ρ*_max_, Δ*ρ*_min_ (e Å^-3^) | 1.36, -0.70 | 1.56, -1.25 | 2.39, -1.50 |

**Table S2.** The tentative assignment of IR and Raman bands (cm^-1^) observed for (TMBM)_2_MnBr_4_ at selected temperatures.^a^

| **IR** | | | **Raman** | **Assignment** |
| --- | --- | --- | --- | --- |
| **10 K** | **300 K** | **400 K** | **RT** |  |
| 3025_vs_, 3023_sh_, 3017_m_, 3013_m_ | 3024_vs_, 3017_sh_ | 3023_vs_ | 3025_s_ | ν_as_CH_3_ |
| 2963_w_, 2957_sh_ | 2964_w_ | 2964_w_ | 2969_s_ | ν_sym_CH_3_ |
| 2939_vw_ | 2934_sh_ | 2930_vw_ | 2940_m_, 2929_sh_ | ν_as_CH_2_ |
| 2912_vw_ |  |  | 2909_w_ | ν_sym_CH_2_ |
| 2896_vw_–2797_vw_ |  |  | 2865_vw_, 2814_w_ | 2δ_as_CH_3_+2δ_as_CH_2_ |
| 1492_sh_, 1486_s_, 1478_vs_, 1474_vs_, 1467_s_, 1465_sh_, 1459_w_, 1456_w_, 1453_sh_ | 1484_vs_, 1474_vs_, 1468_vs_, 1459_m_ | 1484_s_, 1471_vs_ | 1458_w_, 1441_w_ | δ_as_CH_3_+δ_as_CH_2_ |
| 1445_vw_, 1439_vw_, 1434_sh_, 1432_vw_, 1428_sh_, 1421_sh_, 1419_sh_, 1416_w_, 1408_m_, 1405_sh_, 1399_vw_, 1396_vw_, 1386_vw_ | 1433_w_, 1417_sh_, 1410_m_, 1384_vw_ | 1436_sh_, 1411_w_, 1384_vw_ | 1411_w_ | δ_sym_CH_3_+δ_sym_CH_2_ |
| 1321_s_, 1314_sh_, 1314_m_, 1306_w_ | 1320_s_, 1313_sh_, 1305_sh_ | 1317_m_, 1305_sh_ | 1313_vw_ | ωCH_2_ |
| 1280_vw_, 1272_vw_, 1234_vw_, 1230_sh_, 1195_vw_, 1189_vw_ | 1274_vw_, 1231_vw_, 1191_vw_ | 1270_vw_, 1229_vw_, 1191_vw_ | 1191_vw_ | τCH_2_ |
| 1128_sh_, 1125_vw_, 1120_vw_, 1114_vw_, 1110_vw_, 1072_vw_, 1069_vw_, 1067_vw_, 1064_sh_ | 1124_vw_, 1111_vw_, 1068_vw_ | 1124_vw_, 1070_vw_ | 1122_vw_ | ρCH_3_ |
| 982_sh_, 975_m_, 965_m_ | 972_m_, 964_m_ | 971_sh_, 962_m_ | 970_sh_, 964_w_ | ν_as_NC_4_ |
| 916_vs_, 913_sh_, 911_sh_, 905_sh_, 902_sh_, 900_vs_, 891_m_, 887_m_, 878_vw_ | 911_m_, 899_vs_, 892_sh_ | 908_sh_, 896_vs_, 887_sh_ | 895_w_ | ν_sym_NC_4_ |
| 764_sh_, 761_vw_, 757_vw_ | 759_sh_, 757_vw_ | 755_vw_ | 757_w_ | ρCH_2_ |
| 706_w_, 701_m_ | 702_m_ | 702_w_ | 704_vs_ | νCBr |
|  |  |  | 488_w_, 445_vw_ | δ_as_NC_4_ |
|  |  |  | 421_w_, 367_vw_ | δ_sym_NC_4_ |
|  |  |  | 315_m_ | νMnBr_4_ |
|  |  |  | 276_vw_ | τCH_2_Br |
|  |  |  | 206_w_,161_m_, 130_w_ | δMnBr_4_ |
|  |  |  | 78_vs_, 57_vs_ | L+T’ |

^a^ Key: **as**, antisymmetric; **δ**, bending; **L**, libration; **m**, medium; **ν**, stretching **ρ**, rocking; **s**, strong; **sh**, shoulder; **sym**, symmetric; **T’**, translation **τ**, twisting; **vs**, very strong; **vw**, very weak; **w**, weak; **ω**, wagging.

**Table S3**. The calculated structural parameters for [MnBr_4_]^2-^ tetrahedra at 120, 295, and 355 K, and changes of parameters calculated in relation with the values for the intermediate phase, are given in % in parentheses.

|  | **Volume (Å^3^)** | **Distortion index** $\boldsymbol{D}$ **(– ×10^3^)** ^b^  $D=\frac{1}{n}\sum_{i=1}^{n} \frac{\left\vert l_{i}-l_{av} \right\vert}{l_{av}}$,  $n$ – coordination number  $l_{i}$ – distance between central atom and $i$^th^ coordinating atom  $l_{av}$ – average bond length | **Bond angle variance** $\boldsymbol{\sigma}^{\boldsymbol{2}}$ **(°)** ^b^  $\sigma^{2}=\frac{1}{m-1}\sum_{i=1}^{m} \left( \phi_{i}-\phi_{0} \right)^{2}$,  $m$ – no of polyhedral faces × ^3^/_2_  $\phi_{i}$ – $i$^th^ bond angle  $\phi_{0}$ –bond angle for ideal polyhedron |
| --- | --- | --- | --- |
| **HT phase**  **(355 K)** | 7.39 (–5.6) | 6.18 (+364.7) | 12.30 (+149.1) |
| **Intermediate RT phase (295 K)** | **7.83** | **1.33** | **4.94** |
| **LT phase** ^c^  **(120 K)** | 7.93 (+1.3)  8.01 (+2.3)  7.98 (+1.9)  8.02 (+2.4) | 1.64 (+23.3)  7.13 (+436.1)  2.14 (+60.9)  4.46 (+235.3) | 9.61 (+94.5)  5.08 (+2.8)  2.59 (–47.6)  1.69 (–65.8) |

^b^ K. Momma and F. Izumi, VESTA: a three-dimensional visualization system for electronic and structural analysis, *J. Appl. Cryst.* 41 (2008) 653–658, 10.1107/S0021889808012016.

^b^ four nonequivalent MnBr_4_^2-^ units in primitive unit cell.


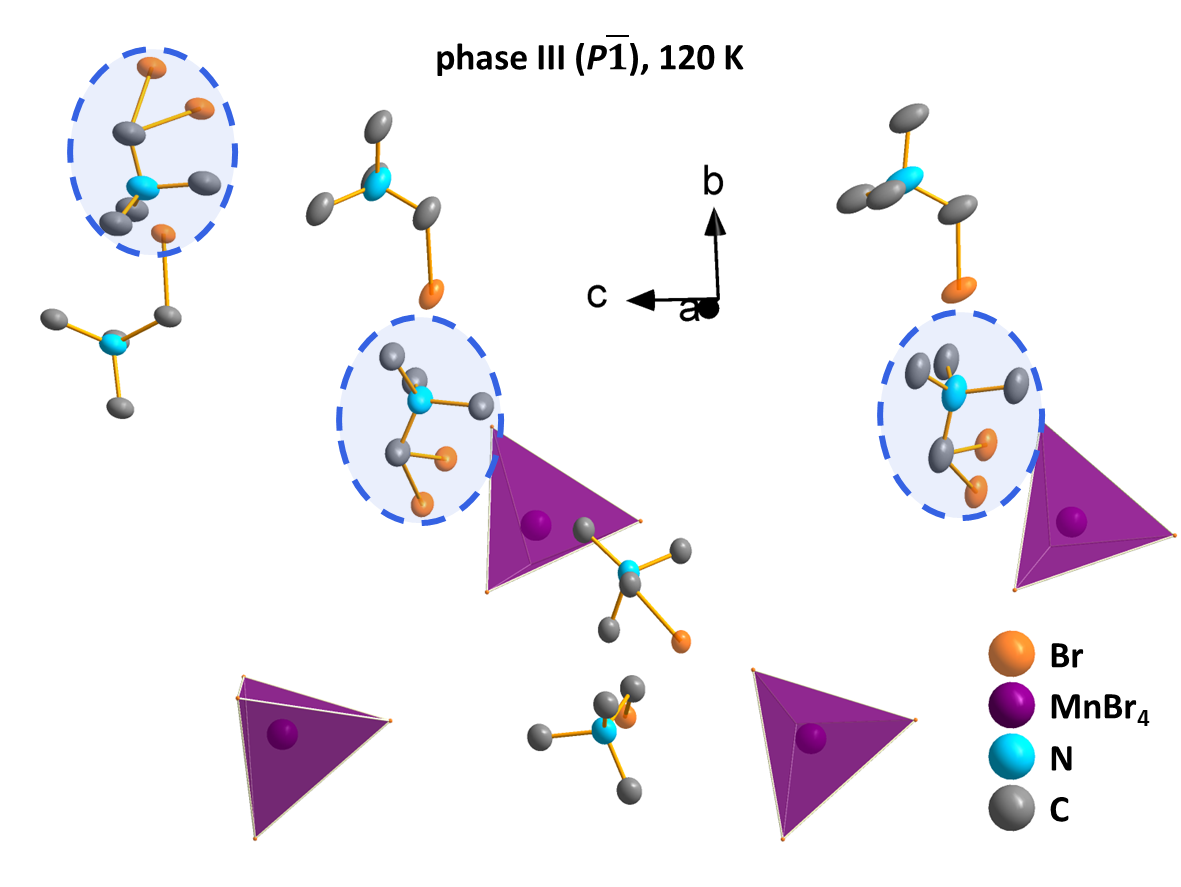


**Figure S1.** Asymmetric unit of (TMBM)_2_MnBr_4_ in phase III derived from the single-crystal XRD structural analysis performed at 120 K, representing [MnBr_4_]^2-^ tetrahedra TMBM^+^ cations (the disordered ones are marked with blue ovals). Hydrogen atoms are removed for clarity.

**Figure S2.** DSC trances for (TMBM)_2_MnBr_4_ for the cooling and heating run.


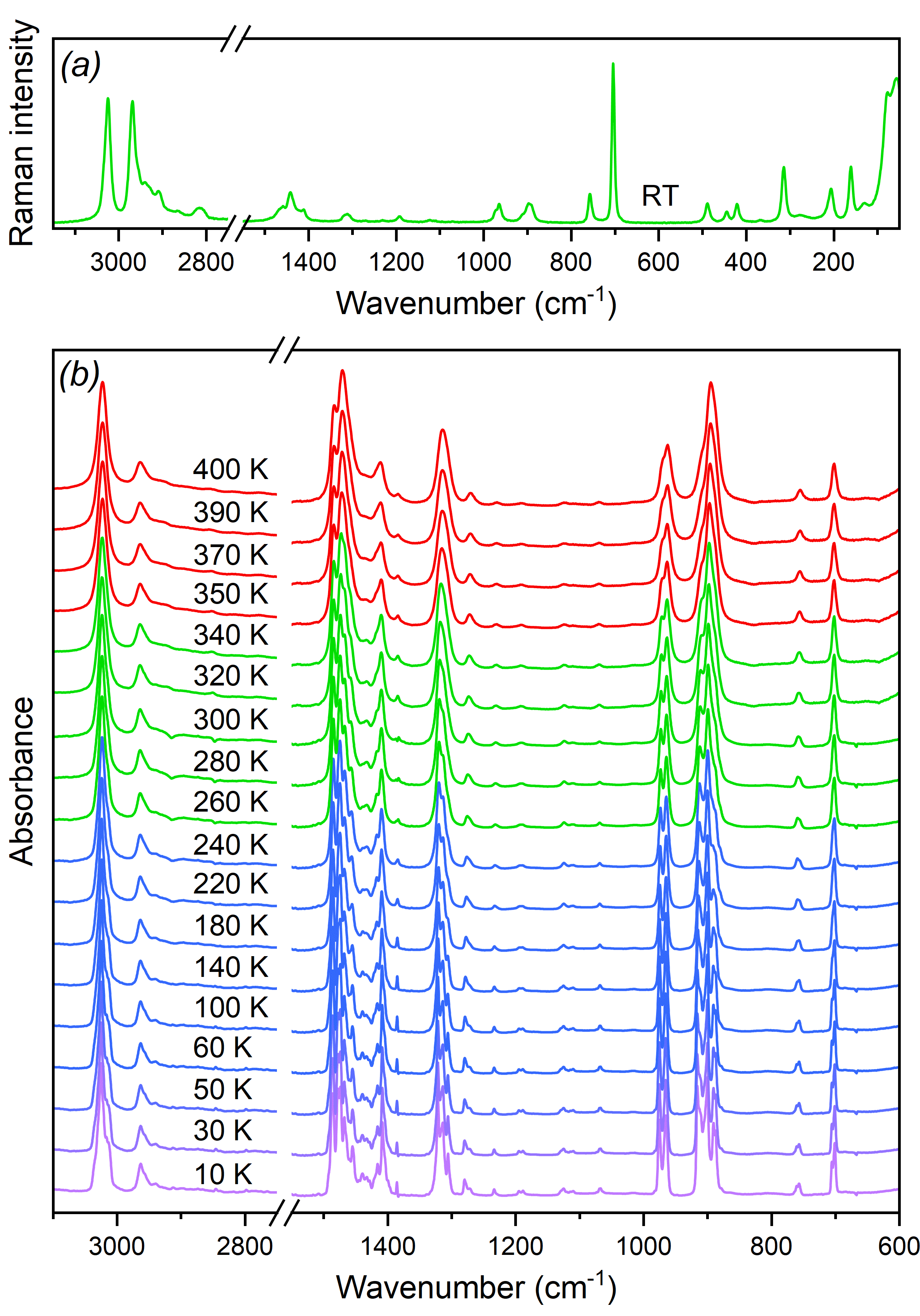


**Figure S3**. The Raman spectrum of (TMBM)_2_MnBr_4_ at room temperature (RT) in the spectral range of 3100–50 cm^-1^ (a) together with temperature evolution (10-400 K) of mid-IR spectra (3100–6000 cm^-1^).


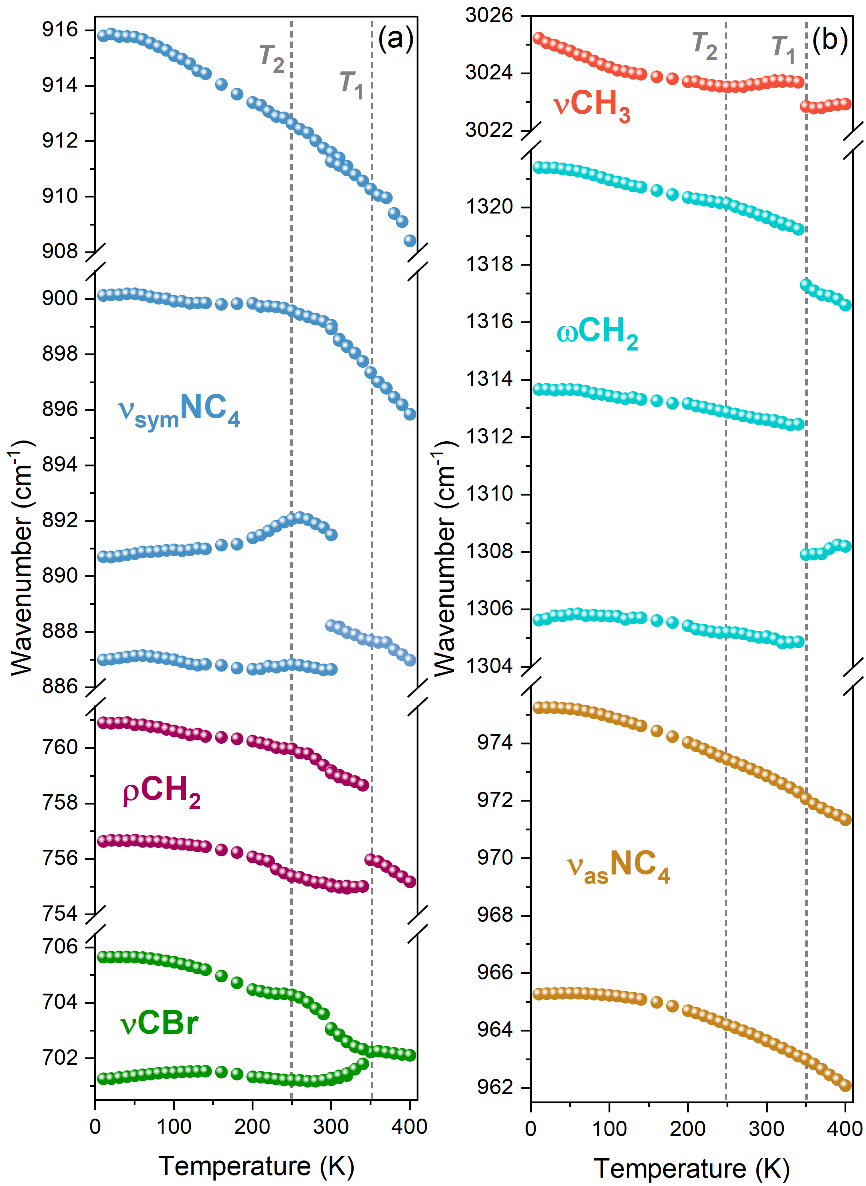


**Figure S4**. The thermal evolution of positions of the selected IR bands; grey vertical lines correspond to PT temperatures obtained from the DSC experiment.


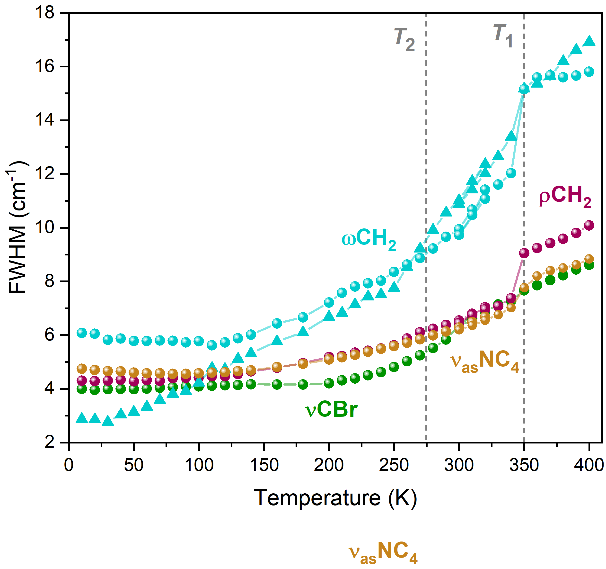


**Figure S5**. The thermal evolution of bandwidths (FWHM, full width at half maximum) of the selected IR bands; grey vertical lines correspond to phase transition temperatures obtained from the DSC experiment.


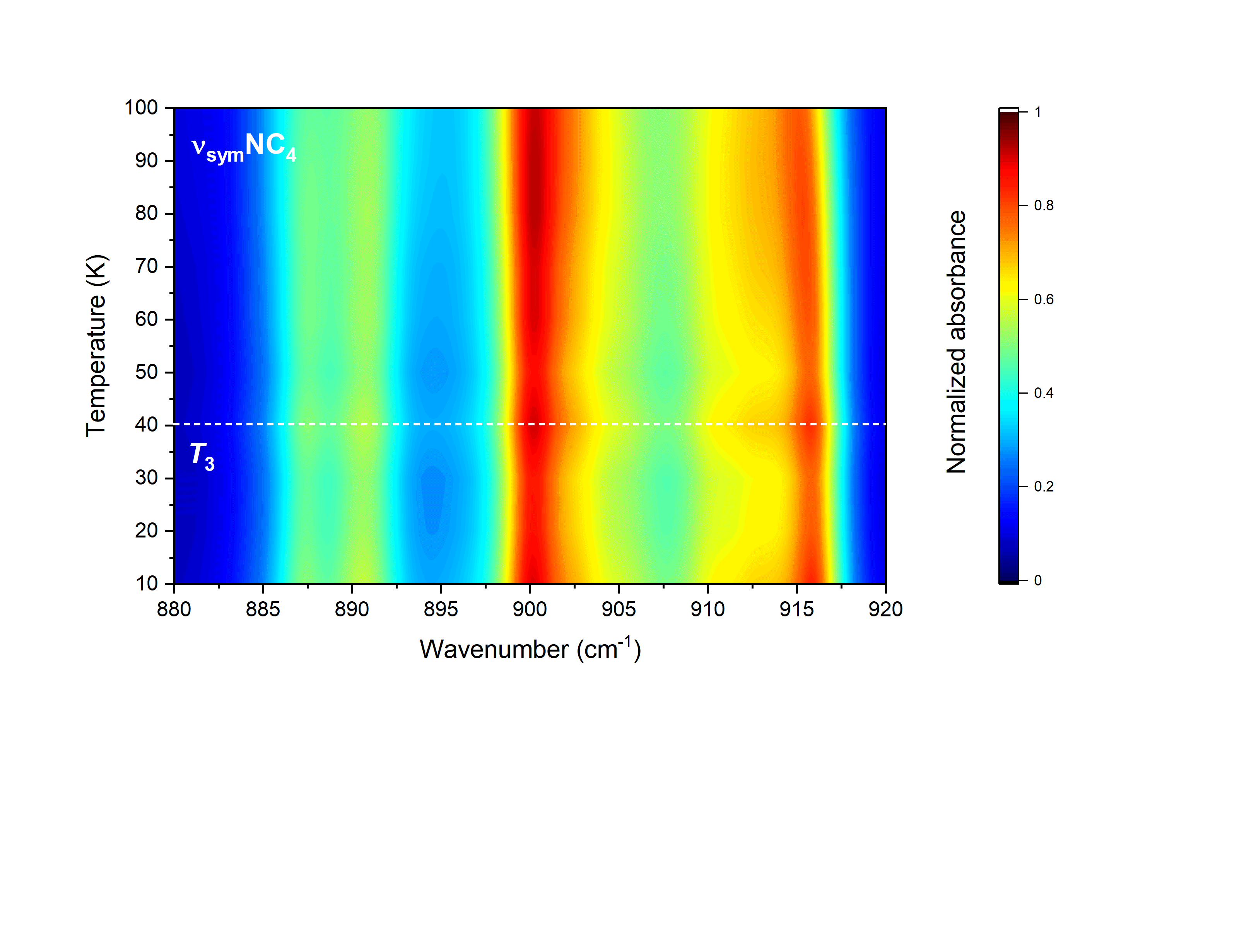


**Figure S6**. An enlargement of color map of absorption magnitudes based on a thermal evolution of IR spectra in the temperature and spectral ranges of 10–100 K and 880–920 cm^-1^, respectively; white horizontal line corresponds to anomaly at T_3_.


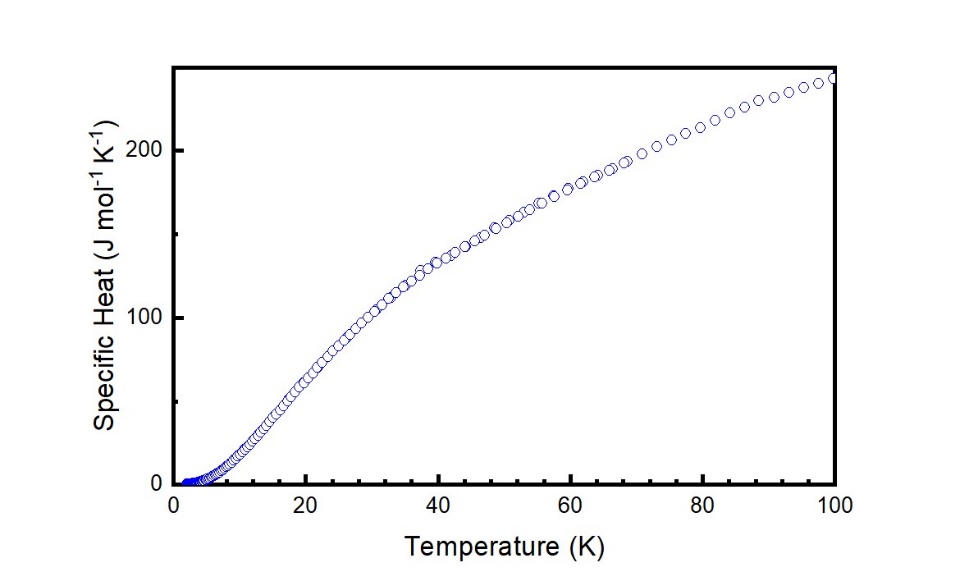


**Figure S7.** The change in heat capacity at low temperatures.


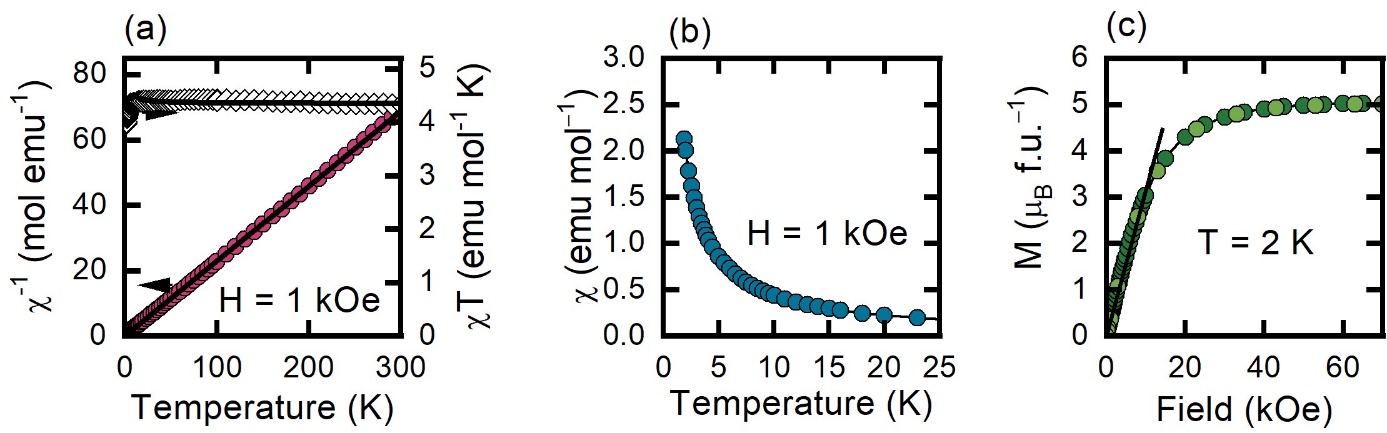


**Figure S8.** (a) Inverse magnetic susceptibility χ^-1^ of (TMBM)_2_MnBr_4_ as a function of temperature T (left axis) and product χT vs T(right axis). thick solid lines are a fit of the Curie-Weiss law to the experimental data. (b) Low-temperature magnetic susceptibility of (TMBM)_2_MnBr_4_ vs. T. (c) Magnetization (M) of the compound (expressed in Bohr magnetons per formula unit) measured with increasing and decreasing H (dark and light symbols), thin solid curve serves as a guide for the eye and thick solid line shows the linear behavior of M(H) at low fields.

**Figure S9.** (a) The UV-Vis absorption spectrum of (TMBM)_2_MnBr_4_ and (b) corresponding Tauc plot.


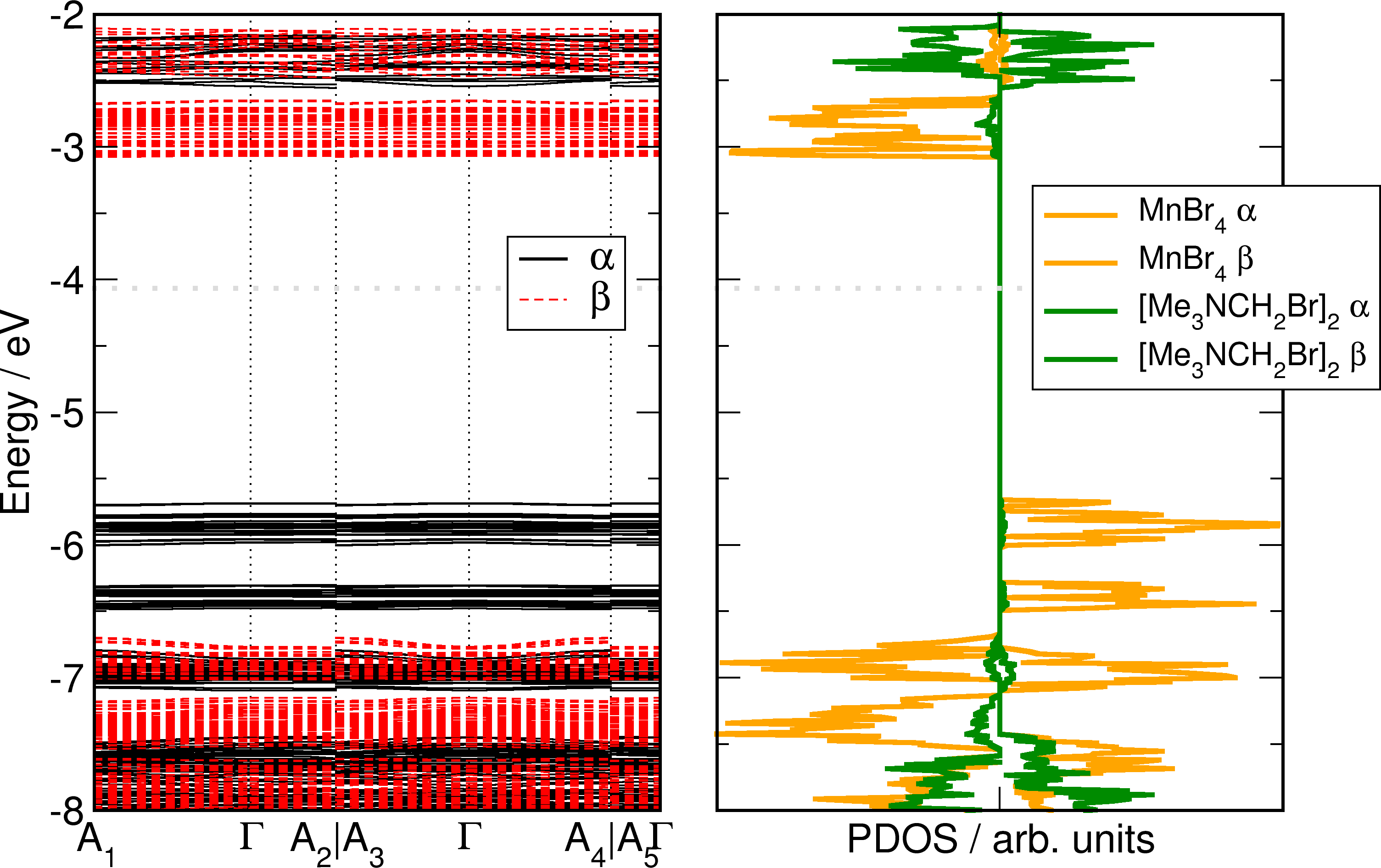


**Figure S10.** Electronic band structures. Fermi level is marked with horizontal grey dotted line.

**Figure S11**. A comparison of optical properties for (TMBM)_2_MnBr_4_ single crystal, PL (460 nm excitation), PLE (monitored at 530 nm), and R spectra at 4K.

**Figure S12**. A comparison of PL spectra measured as a function of excitation wavelength.


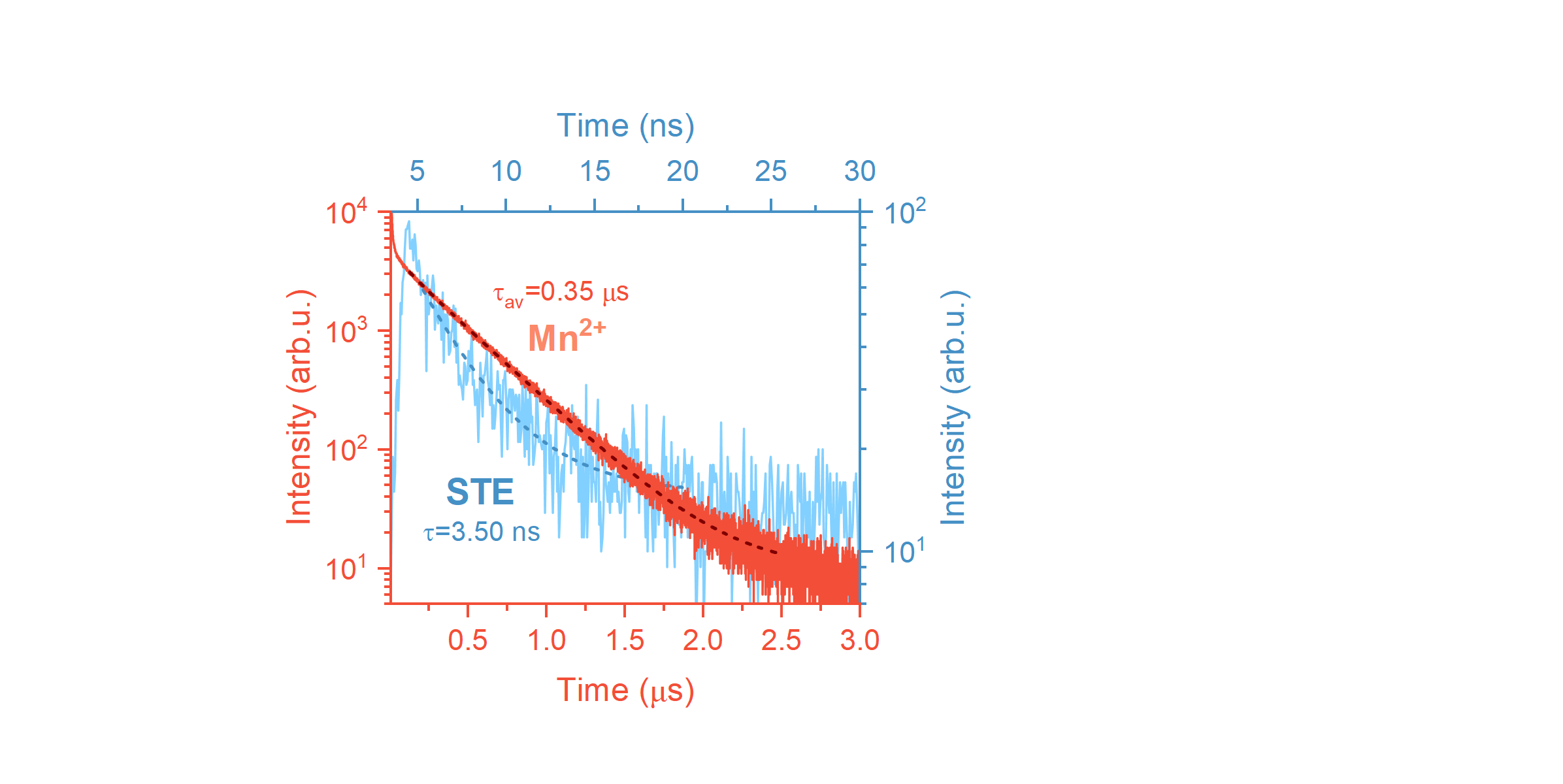


**Figure S13**. The PL decay curves for Mn^2+^ ions at 540 nm (red) and for STE at 560 nm (blue); the dash lines represent fit curves.


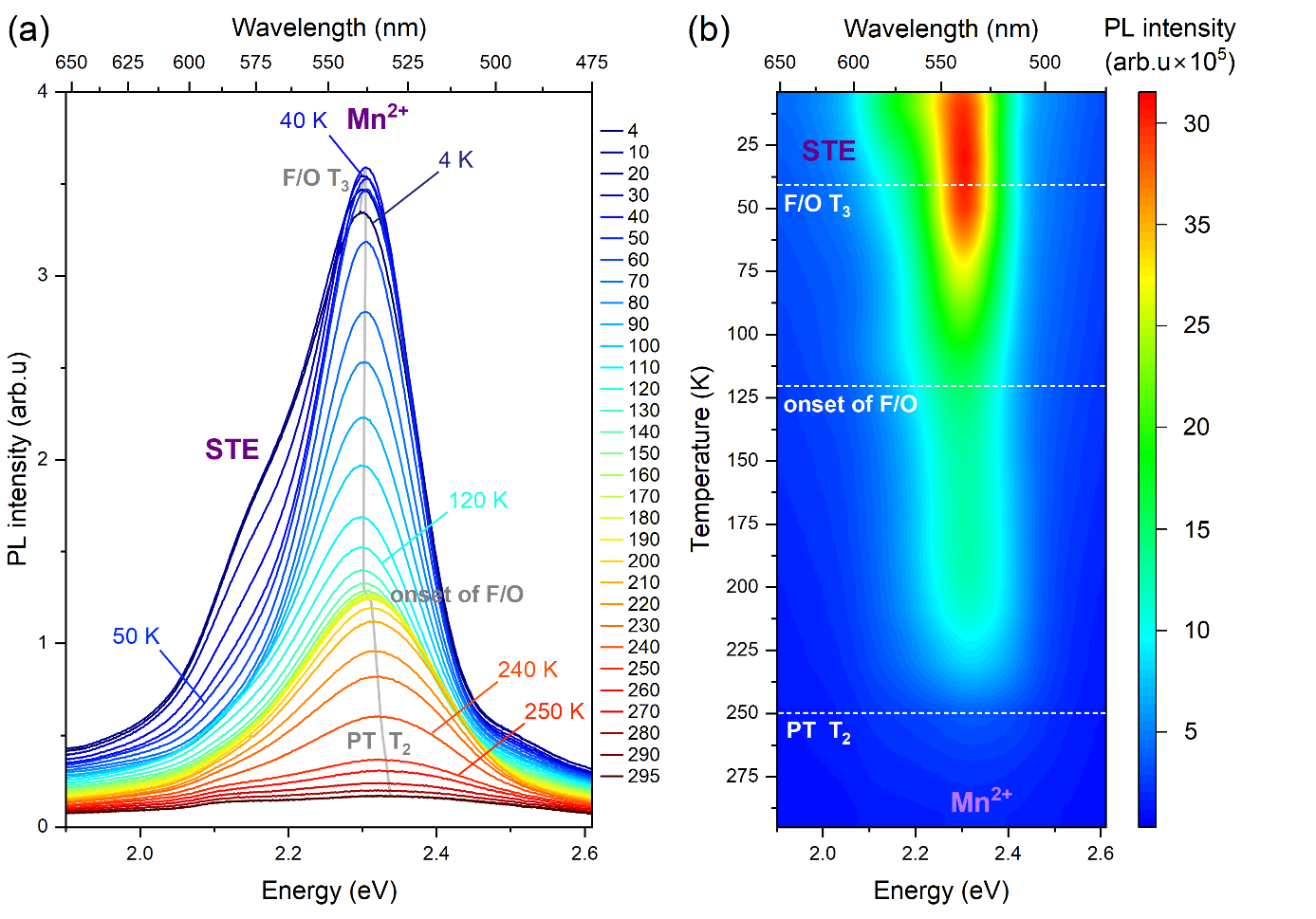


**Figure S14**. The evolution of PL spectra as a function of temperature (a) and their presentation as a colour map of intensity; the grey line in (a) is added to follow the changes; the horizontal lines in (b) correspond to the temperature of phase transition (PT) at T_2_, the anomaly near 110-120 K labelled as an onset of cation freezing (F) or ordering (O), and to anomaly at T_3_.

**Figure S15**. (a) PL spectrum of (TMBM)_2_MnBr_4_ measured at 20 K (black curve). To reproduce the PL spectrum in the whole range two Gauss-profiled components were used. Red and blue solid lines represent Mn^2+^ and STE emissions, respectively. (b) Temperature-induced changes in the intensities of both PL peaks. (c) Activation energies extracted by fitting the Arrhenius model.


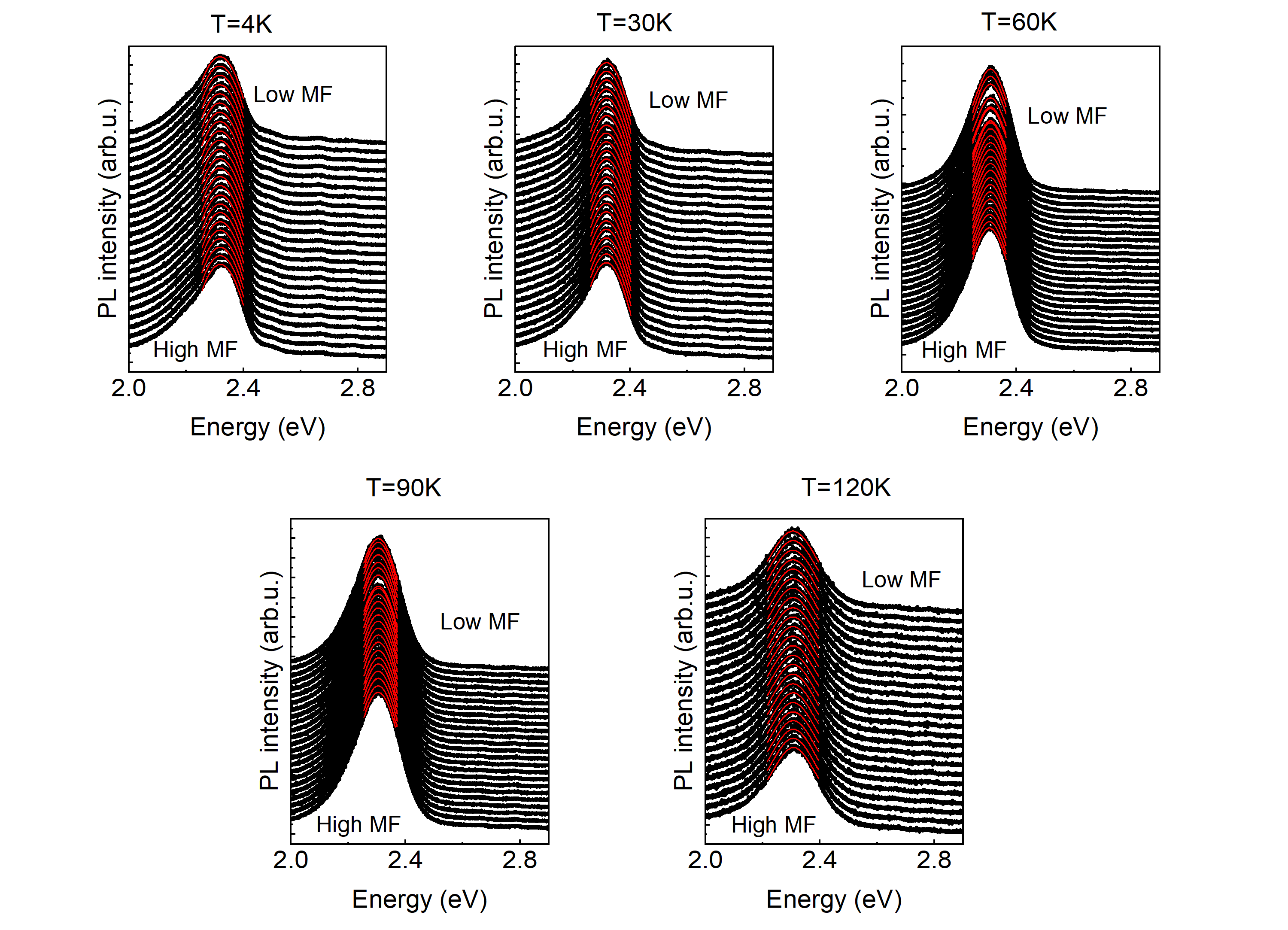


**Figure S16**. The evolution of PL spectra in a magnetic field (black lines) at different temperatures fitted with Gaussian functions (red lines) for (TMBM)_2_MnBr_4_ single crystal.
